# The NR3C2-SIRT1 signaling axis promotes autophagy and inhibits epithelial mesenchymal transition in colorectal cancer

**DOI:** 10.1038/s41419-025-07575-3

**Published:** 2025-04-14

**Authors:** Feng Li, Xing Wan, Zhigui Li, Liming Zhou

**Affiliations:** 1 Department of Pharmacology, West China School of Basic Science and Forensic Medicine, Sichuan University, Chengdu, China; 2 Department of General Surgery, West China Hospital, Sichuan University, Chengdu, China

**Keywords:** Tumour biomarkers, Metastasis, Colorectal cancer, Epithelial-mesenchymal transition

## Abstract

Colorectal cancer (CRC) is one of the most aggressive and lethal cancers with a complex pathogenesis, there is an urgent need to find new drug therapeutic targets. This study highlights the important role of the NR3C2-SIRT1 signaling axis in the metastasis mechanism of CRC. Our findings revealed that the expression of NR3C2 in CRC tissues was lower than that in adjacent non-cancerous tissues, and was negatively correlated with N stage by bioanalysis, IHC, western blot and qRT-PCR. NR3C2 overexpression / knockdown can significantly inhibit / promote the migration and invasion of CRC cells, at the same time inhibit / promote EMT. Mechanically, the regulatory molecule SIRT1 was identified by RNA-seq, bioinformatics analysis, western blot and ChIP. SIRT1 was also involved in the metastasis process of CRC, and NR3C2 was found to regulate the expression of LC3B and SQSTM1/p62 in a SIRT1-dependent manner. Therefore, NR3C2 forms a signaling axis with SIRT1, which can directly promote autophagy and inhibit EMT process in vivo and in vitro. Collectively, our findings suggest that NR3C2 - SIRT1 signal axis promote autophagy and inhibit EMT, ultimately inhibits lung metastasis of CRC.

## Introduction

Colorectal cancer (CRC) is the third leading cause of cancer-related deaths worldwide, and it is on the rise in individuals under the age of 50 [1]. With a high incidence rate, CRC is often difficult to detect in its early stages, and there is a significant likelihood of metastasis at the time of initial diagnosis. Therefore, metastatic colorectal cancer (mCRC) remains a deadly disease, with a 5-year survival rate of approximately 14% [2]. While early-stage CRC can be cured through surgical treatment without the need for adjuvant chemotherapy, the presence of disseminated cancer cells, including treatment-resistant metastatic cells, hinders the eradication of mCRC. To address this gap, various research efforts are focused on further elucidating the molecular mechanisms of CRC metastasis and recognizing the limitations of current therapeutic interventions.

In the latest research, attention has been drawn to the involvement of autophagy in the development of CRC [3, 4]. Autophagy is a highly conserved catabolic process that mediates the degradation of harmful or dysfunctional cellular components, such as invasive pathogens, aging proteins, and organelles [5, 6]. The role of autophagy in tumors is complex and can either promote or inhibit growth depending on the tumor stage, the tumor’s microenvironment, and the influence of tumor metabolites [7]. Studies have confirmed that autophagy is involved in several critical biological pathways essential for metastasis, including migration, invasion, epithelial-mesenchymal transition (EMT), adaptation to nutrient deprivation, and response to hypoxia [8–10].

In our previous research, we identified a pivotal member gene within the nuclear receptor superfamily - Nuclear Receptor Subfamily 3 Group C Member 2 (NR3C2). Positioned on human chromosome 4q31.23, NR3C2 encodes the mineralocorticoid hormone receptor (MR), a crucial stress-related gene receptor. Our previous studies have unveiled NR3C2’s profound impact on CRC cells proliferation, we demonstrated that NR3C2 inhibited the proliferation of CRC cells by inhibiting glucose metabolism and phosphorylation of AMPK [11]. Recent international and domestic studies have highlighted the anti-cancer role of NR3C2 in various tumor types [12–19]. However, there has been limited research on the molecules regulated by NR3C2.

SIRT1, a member of the deacetylase family, is intricately linked to sirtuins [20]. Previous studies have shed light on SIRT1’s role in promoting autophagy by translationally modifying autophagy initiation proteins (ATG5/7/8 and microtubule-associated protein light chain 3 (LC3)) [21–24], as well as deacetylation-dependent activation of autophagy-related transcription factors (TFEB and FoxO) [25, 26]. So SIRT1 is a regulator of autophagy. Notably, the intricate relationship between AMPK and autophagy has been reported [27]. However, the enigmatic connection between NR3C2 and autophagy remains unexplored, and the mechanisms through which NR3C2 orchestrates the EMT process via autophagy remain elusive. Therefore, On the basis of previous research, this study explored the relationship between NR3C2 and autophagy, and found that NR3C2 overexpression promoted autophagy in CRC cells for the first time. We discussed that the regulation of SIRT1 by NR3C2 was found for the first time. The combination of SIRT1 with autophagy inhibitors and agonists confirmed that the NR3C2-SIRT1 axis promotes autophagy, thereby inhibiting EMT process.

## Materials and methods

### Cells culture

All cells were cultured in the Dulbecco’s modified Eagle’s medium (DMEM) supplemented with 10% fetal bovine serum (FBS), 100 U/mL penicillin, and 100 μg/mL streptomycin. Cells were cultured in a humidified incubator with 5% CO_2_ at 37 °C.

### Patients and tissue samples

All CRC tumor tissues and adjacent non-cancerous tissues (5.0 cm beyond the tumor tissues) were collected at the Department of Surgery, West China Hospital, Sichuan University between 2019 and 2021. Tumor tissues were taken from CRC tumor lesions, adjacent non-cancerous tissues were taken from normal mucosal tissues (5.0 cm beyond the tumor tissues). For example, patients with sigmoid colon cancer, tumor tissues were taken from sigmoid colon tumor lesion, adjacent non-cancerous tissues were taken from normal mucosal tissues of sigmoid colon (5.0 cm beyond the tumor tissues). As patients with rectal cancer, tumor tissues were taken from rectal tumor lesion, adjacent non-cancerous tissues were taken from normal mucosal tissues of rectum (5.0 cm beyond the tumor tissues). A total of 246 cases were collected in this study. The collection and use of human tissues for this study were approved by the Ethics Committee of West China Hospital. Written informed consent was obtained from patients before sampling. TNM staging was based on the 8th edition of AJCC [28].

### Cells transfection

The shRNA plasmid of NR3C2 and negative control shRNA were commercially obtained from hanheng (shanghai, China). Sequences of NR3C2 shRNA were listed in Table S1. Two distinct vectors were utilized in this study: hU6-MCS-CMV-ZsGreen 1-PGK-Puro (PHY-310, labeled with sh310) and CMV-ZsGreen1-MCS-PGK-Puromycin (PHY-502, labeled with sh502). NR3C2 overexpression uses lentivirus infection, the construction mode was consistent with the previous [11].

For siRNA transfection, CRC cells were seeded in 6-well plates, and transfected with Lipofectamine^TM^ RNAiMAX transfection reagent (13778030, Thermo Fisher Scientific, USA) according to the manufacturer’s protocol. Sequences of SIRT1 siRNA were listed Table S2.

### RNA isolation and quantitative real-time PCR (qRT-PCR)

The transfected cells were lyzed with Trizol Reagent (Invitrogen, USA) according to the manufacturer’s instructions. The concentration and purity of RNA were detected by instrument (NanoDrop One, Thermo Scientific, USA), reverse transcription was performed using a PrimeScript RT reagent Kit (Takara, Dalian, China). For qPCR analysis, cDNA was amplified with a SYBR Premix Ex Taq (Takara) kit by using an ABI 7500 Real-time PCR system. The relative gene expression was calculated by the 2^−△△Ct^ method. For NR3C2 qRT-PCR, the primers for NR3C2 (Forward: GAACACGCCCTTGAGATCAT, Reverse: AGAGGAGTTCCCTGGGTGAT), GAPDH (Forward: TGAACGGGAAGCTCACTGG, Reverse: TCCACCACCCTGTTGCTGTA) and SIRT1 (Forward: TGCTGGCCTAATAGAGTGGCA, Reverse: CTCAGCGCCATGGAAAATGT) were used.

### Cells migration and invasion assays

The matrix glue was thawed on ice, diluted with DMEM (matrix glue: DMEM = 1:8, this step was omitted in the migration experiment), and then placed in a 37 °C, 5% CO_2_ incubator for 3 h. The CRC cells were digested and counted, 200 μL cells were added to each chamber (4 × 10^4^ cells were suspended in 1% DMEM), and then cultured at 37 °C in 5% CO_2_ incubator for 24 h. The cells were fixed with 5% paraformaldehyde for 30 min, and then stained with 5% crystal violet for 30 min. Finally, the cells were observed under an inverted microscope followed by imaging and counting (Leica, Germany).

### Wound healing assay

Three straight lines were drawn on the back of the 24-well plate, and the appropriate amount of CRC cells were cultured in DMEM with 10% FBS overnight. Draw a line vertically with a 200 μL pipette tip and clean the fallen cells with PBS. Culture with DMEM without FBS for 24 h. The crawling positions of cells at 0 h and 24 h time points were recorded under an inverted microscope (Leica, Germany), and the crawling area analyzed by Image J.

### Western blot

The cells were lysed by the RIPA buffer (Beyotime, China) containing PMSF (Solarbio, China) for 30 min, and the cells were fully lysed with vortex oscillation every 5 min. The cells were centrifuged at 4 °C for 13000 g for 10 min, the supernatant was separated, appropriate amount of loading buffer (Beyotime, China) was added, and the samples were boiled at 100 °C for 10 min. Supernatant was added to SDS-PAGE gel and transferred to NC film (PALL, USA). After the transfer membrane was completed, it was closed in a shaker with 5% skim milk for 2 h, incubated with specific primary antibody at 4 °C overnight, and then incubated with goat anti-Rabbit IgG or goat anti-mouse IgG antibody (SAB, SUA) at room temperature for 1.5 h. Finally, protein expression was detected using a chemiluminescence detection system (CLINX 6200Touch, China), and grayscale was analyzed by image J. Specific antibodies information was showed in Table S3.

### RNA-sequencing (RNA-seq)

Total RNA was extracted using RNAprep Pure Plant Plus Kit (DP441, TIANGEN) and purified by RNAClean XP Kit (A63987, Beckman Coulter) and RNase-Free DNase Set (79254, Qiagen). Libraries were pooled and sequenced using the Illumina NovaSeq machine as 150-bp paired-end sequencing reads. Raw reads were trimmed using Fastp v0.20.0, removing low-quality reads and removing reads with size inferior to 50 bp (--length_required 50). Then, the reference genome and gene model annotation files were downloaded from the genome website directly (ftp://ftp.solgenomics.net/tomato_genome/assembly/build_4.00/). Clean data were mapped to the reference genome using HISAT2 v 2.1.0. Next, the mapped reads of each sample were assembled using StringTie v1.3.6 in a reference-based approach. All RNAs were quantified as FPKM (Fragments Per Kilobase Million Mapped Reads) by HTSeq(v0.13.5,http://htseq.readthedocs.io/en/release_0.9.1).

### Bioinformatics analysis

The relationship between the expression of NR3C2, SIRT1, and clinicopathological characteristics was validated using the public website GEPIA (http://gepia.cancer-pku.cn/) and the TCGA database. Additionally, TIMER2.0 (cistrome.shinyapps.io/timer) was employed for analyzing the correlation between NR3C2 and SIRT1.

### Immunohistochemical (IHC)

The expressions of NR3C2 and SIRT1 proteins were determined by IHC in the CRC tumors and adjacent non-cancerous tissues. The specific operation process is consistent with previous research [29]. Paraffin-embedded biopsies were incubated with primary antibodies (NR3C2:1:8000; Abcam; SIRT1:1:100, Abcam).

### Animal experiments

A total of 40 nude mice (4–6 weeks old) were purchased from Beijing Vital River Laboratory Animal Technology, randomly divided into the following four groups with ten mice each, HCT116 vector, HCT116 NR3C2, RKO vector and RKO NR3C2 cells were adjusted to a concentration of 4*10^6^ cells in 100 μL and injected by tail vein. The status of mice was observed every day, and the mice were sacrificed after 8 weeks, and the lung tissues were fixed, and hematoxylin-eosin (HE) and IHC staining were performed. All animal experiments were approved by the Institutional Animal Care and Use Committee of Sichuan University.

### Immunofluorescence

10 μL of DMEM were first added to a 24-well plate. Subsequently, the cell slides were placed, allowing them to adhere to the plate. HCT116 and SW620 cells were then seeded into the 24-well plate. After 24 h, 4% paraformaldehyde was added to the wells for fixation for 15 min. Following fixation, cells were incubated with 0.2% Triton X-100 for 10 min, followed by a 30-min incubation with blocking solution. Primary antibodies (NR3C2: 1:1000, Abcam; SIRT1: 1:1000, HUABIO) from different species were applied and incubated at 4 °C overnight. For the secondary antibody incubation, species-specific secondary antibodies (Donkey anti-mouse IgG(H + L) secondary antibody, Alexa Fluor 488; Donkey anti-rabbit IgG(H + L) secondary antibody, Alexa Fluor 594; Thermo Fisher, USA) were applied and incubated at room temperature for 1 h. DAPI was used for nuclear staining for 5 min. The slides were then inverted onto new coverslips containing a small amount of antifade mounting medium and labeled appropriately. Imaging was conducted using laser confocal microscopy (ZEISS, Germany).

### Chromatin immunoprecipitation (ChIP)

The ChIP assay was used to verify the transcriptional factor NR3C2 binding at the promoter of the SIRT1. The HCT116 cells and SW620 cells were fixed with formaldehyde, and cross-linking was performed by adding glycine. Cell lysates were incubated with antibodies (anti-SIRT1 antibody or anti-NR3C2 antibody or control IgG), followed by incubation with protein A/G magnetic beads (MCE, USA) at 4 °C overnight. The bound DNA-protein mixtures were eluted, and cross-linking was reversed after several washes, the mixtures were further measured by qRT-PCR. The test was repeated three times.

### Statistical analysis

All data from in vitro experiments were obtained from three independent experiments. The data were analyzed by one-way ANOVA or Student’s t test, and statistical analyses were performed using SPSS21.0 (SPSS, Chicago, IL, USA) and GraphPad Prism 8.0 (GraphPad software, La Jolla, CA, USA).

## Results

### NR3C2 inhibits CRC cells migration as an anti-oncogene

**Fig. 1:**
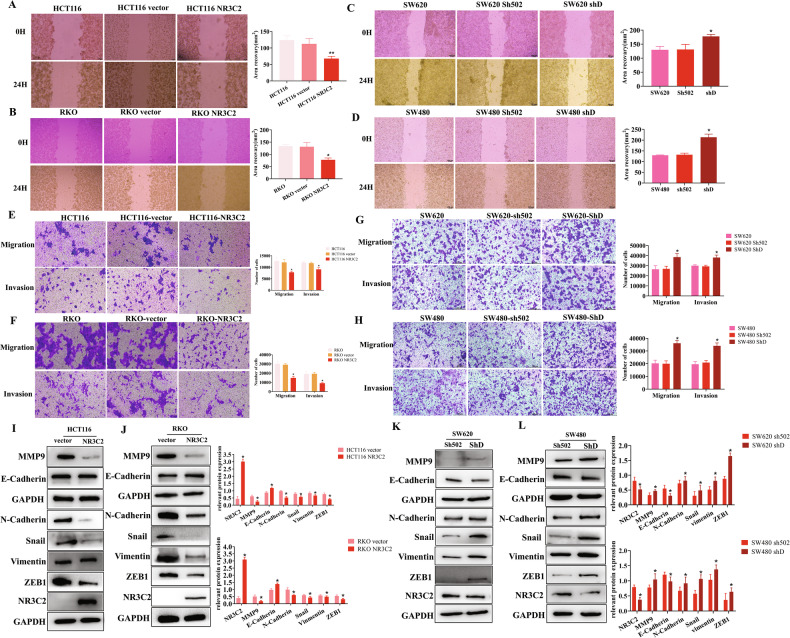
NR3C2 inhibits CRC cells migration as an anti - oncogene. **A** Wound healing assays in HCT116 cells, **P < 0.01; **B** wound healing assays in RKO cells, *P < 0.05; **C** wound healing assays in SW620 cells, *P < 0.05; **D** wound healing assays in SW480 cells, *P < 0.05; **E** migration and invasion experiment in HCT116 cells, *P < 0.05; **F** migration and invasion experiment in RKO cells, *P < 0.05; **G** migration and invasion experiment in SW620 cells, *P < 0.05; **H** migration and invasion experiment in SW480 cells, *P < 0.05; **I** Western blot detection of EMT-related proteins in HCT116 cells, *P < 0.05; **J** Western blot detection of EMT-related proteins in RKO cells, *P < 0.05. **K** Western blot detection of EMT-related proteins in SW620 cells, *P < 0.05; **L** Western blot detection of EMT-related proteins in SW480 cells, *P < 0.05.

Seven different CRC cells lines were detected for the expression of NR3C2. The expression of NR3C2 in each cell line was assessed by qRT-PCR and Western blot (Fig. S1A, B). HCT116 and RKO cells were selected for NR3C2 overexpression, while SW620 and SW480 cells were subjected to knockdown, combining the mRNA and protein expression. Transfection efficiency was observed under a fluorescence microscope, qRT-PCR and western blot after transfection of HCT116 and RKO cells (Fig. S1C–E). SW620 and SW480 cells were subjected to NR3C2 knockdown using ShRNA. Transfection efficiency was observed under a fluorescence microscope, qRT-PCR and western blot after transfection (Fig. S1F–H). Wound healing assays, transwell migration and invasion experiments showed that when NR3C2 was overexpressed, the number of migration and invasion declined in CRC cells (Fig. 1 A B, E, F). when NR3C2 was knocked down, the number of migration and invasion increased in CRC cells (Fig. 1C, D, G, H). Following assessment of NR3C2’s impact on the phenotype through wound healing assays and transwell migration and invasion assays, we detected the EMT-related proteins. When NR3C2 was overexpressed, the expression of E-cadherin increased, while the expression of N-cadherin, MMP9, Snail, vimentin, and ZEB1 decreased, all showing statistical significance. Consistent results were observed in HCT116 and RKO cells (Fig. 1I, J). A decrease in E-cadherin and an increase in the expression of N-cadherin, MMP9, Snail, vimentin, and ZEB1 when NR3C2 was knocked down. These changes were statistically significant, and the experimental results were consistent in both SW620 and SW480 cells (Fig. 1K, L). This demonstrates that NR3C2 inhibits CRC cells migration as an anti – oncogene.

### NR3C2 forms a signal axis with SIRT1 in CRC cells

**Fig. 2:**
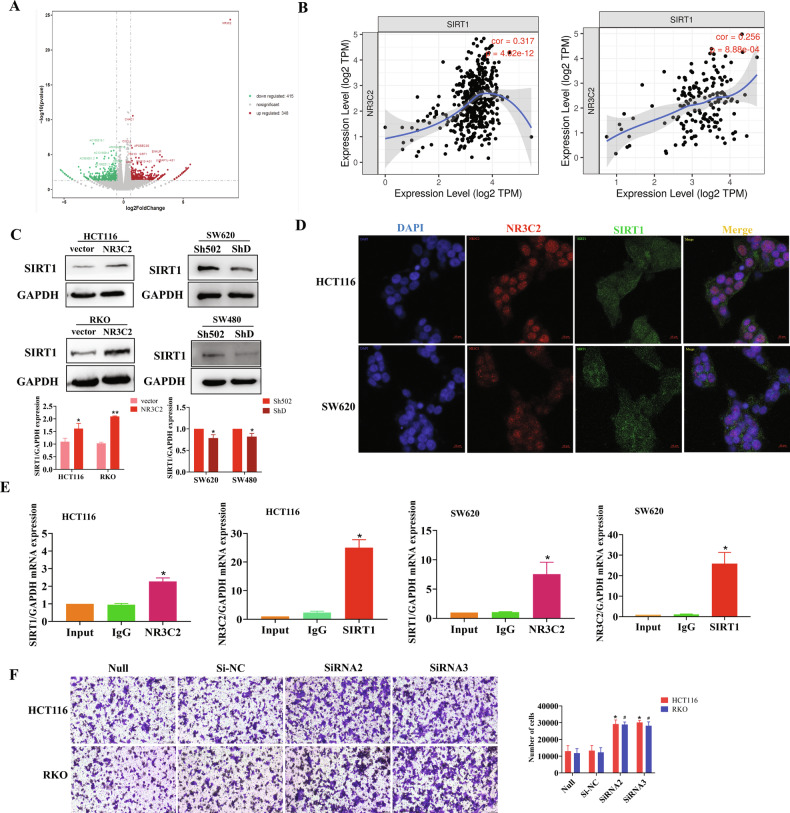
NR3C2 and SIRT1 form a signal axis. **A** Volcano plot of downstream regulatory gene after NR3C2 overexpression. **B** TIMER prediction of the relationship between NR3C2 and SIRT1. left: COAD; right: READ; **C** Western blot detection of SIRT1 expression after NR3C2 overexpression and NR3C2 knockdown, *P < 0.05, **P < 0.01; **D** Fluorescence double staining to detect the expression of NR3C2 and SIRT1 (Blue: DAPI; Red: NR3C2; Green: SIRT1). Upper: HCT116, lower: SW620; **E** ChIP assay to detect the relationship between NR3C2 and SIRT1. *P < 0.05. **F** Transwell migration experiment validating that siSIRT1 increases the decrease in cells migration. Upper: HCT116, lower: RKO, right: statistical chart, *P < 0.05(HCT116), #*P* < 0.05(RKO).

Multiple genes regulated by NR3C2 overexpression were identified by RNA-seq (Fig. 2A), and the relationship between these genes and NR3C2 was predicted by TIMER, a positive correlation between NR3C2 and SIRT1 was indicated (Fig. 2B). Consequently, we examined the co-expression of these two proteins within the same cell. When NR3C2 overexpression, SIRT1 expression increased, whereas NR3C2 knockdown led to a decrease in SIRT1 expression (Fig. 2C). To further explore the molecular relationship between NR3C2 and SIRT1, we performed fluorescence double staining, revealing predominant nuclear expression of NR3C2 (red) and dual expression of SIRT1 in the cell membrane and nucleus (green) (Fig. 2D). In order to verify the combination of NR3C2 and SIRT1, we carried out ChIP experiment, and we found that the combination of NR3C2 protein and a SIRT1 DNA fragment in the HCT116 cells and SW620 cells (Fig. 2E). Of course, we also used siRNA for rescue experiments, qRT-PCR and Western blot detected transfection efficiency, siRNA2 and siRNA3 were selected for follow-up experiments (Fig. S2A, B). When SIRT1 was silenced, the migration number of HCT116 cells increased, as did RKO cells (Fig. 2F).

### NR3C2-SIRT1 axis regulates CRC cells EMT

**Fig. 3:**
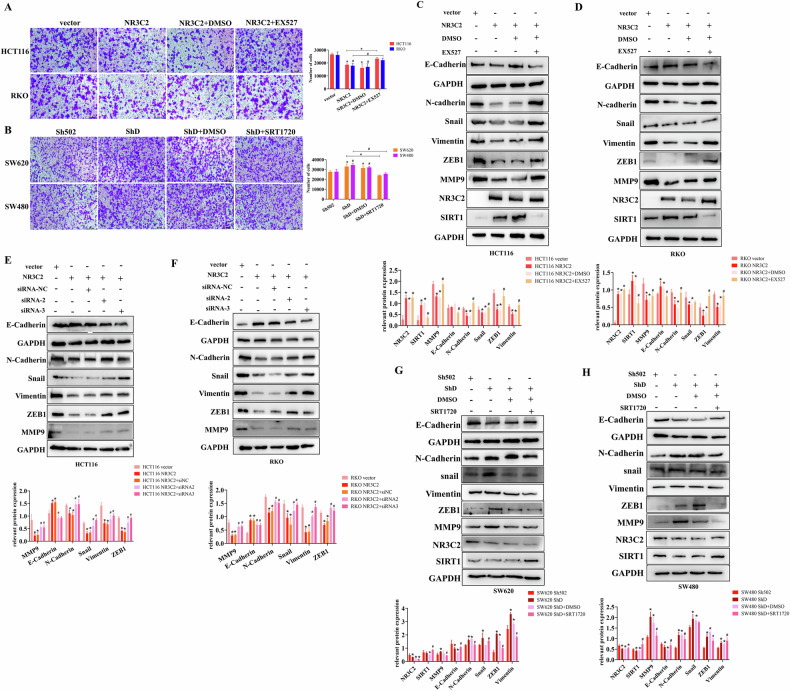
NR3C2-SIRT1 axis regulates CRC cells EMT. **A** Transwell migration experiment validating that EX527 increases the decrease in cells migration caused by NR3C2 overexpression. Upper: HCT116, lower: RKO, right: statistical chart, *P < 0.05(HCT116), #*P* < 0.05(RKO); **B** Transwell migration experiment validating that SRT1720 decreases the increase in cells migration caused by NR3C2 knockdown. Upper: SW620, lower: SW480, right: statistical chart, *P < 0.05(SW620), #*P* < 0.05(SW480); **C** Western blot detection of the impact of EX527 on EMT-related proteins after NR3C2 overexpression in HCT116 cells., *P < 0.05 vs. vector, #*P* < 0.05 vs. NR3C2 + DMSO; **D** Western blot detection of the impact of EX527 on EMT-related proteins after NR3C2 overexpression in RKO cells *P < 0.05 vs. vector, #*P* < 0.05 vs. NR3C2 + DMSO; **E** Western blot detection of the impact of siSIRT1 on EMT-related proteins after NR3C2 overexpression in HCT116 cells. *P < 0.05 vs. vector, #*P* < 0.05 vs. NR3C2 + siNC; **F** Western blot detection of the impact of siSIRT1 on EMT-related proteins after NR3C2 overexpression in RKO cells. *P < 0.05 vs. vector, #*P* < 0.05 vs. NR3C2 + siNC; **G** Western blot detection of the impact of SRT1720 on EMT-related proteins after NR3C2 knockdown in SW620 cells. *P < 0.05 vs. Sh502, #*P* < 0.05 vs. ShD + DMSO; **H** Western blot detection of the impact of SRT1720 on EMT-related proteins after NR3C2 knockdown in SW620 cells. *P < 0.05 vs. Sh502, #*P* < 0.05 vs. ShD + DMSO.

To further validate this relationship, we conducted rescue experiments using the SIRT1 activator SRT1720 and SIRT1 inhibitor EX527. With increasing EX527 concentration, SIRT1 expression decreased, while NR3C2 expression remained constant (Fig. S3A, B). Conversely, as the concentration of SRT1720 increased, SIRT1 expression rose, with NR3C2 expression remaining unaffected (Fig. S3C, D). This indicates that SIRT1 is downstream of NR3C2. Cells stimulated with EX527 exhibited enhanced migration capacity, whereas cells stimulated with SRT1720 showed reduced migration capacity (Fig. S3E, F). When NR3C2 was overexpressed, the number of migrating cells decreased. but the number of migrating cells increased when added EX527 (Fig. 3A). Conversely, when NR3C2 was knocked down, the number of migrating cells increased, but this effect was mitigated upon addition of the SRT1720 (Fig. 3B). Consistently, EMT-associated proteins are restored when EX527 is added. (Fig. 3C, D). When NR32C was overexpressed, EMT associated proteins are restored when siRNA is added (Fig. 3E, F). Consistently, When NR32C was knocked down, EMT-associated proteins are restored when SRT1720 is added (Fig. 3G, H). All the results were statistically significant. Therefore, all results indicated that NR3C2-SIRT1 axis regulates CRC cells EMT.

### NR3C2 promotes autophagy and regulates EMT in CRC cells

**Fig. 4:**
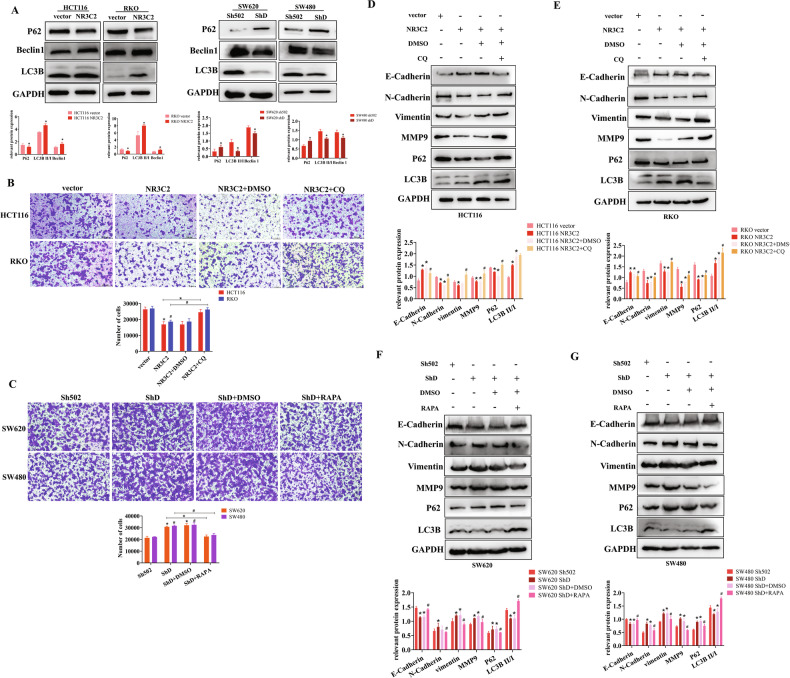
NR3C2 promotes autophagy and regulates EMT in CRC cells. **A** Western blot detection of changes in autophagy-related proteins after NR3C2 overexpression and NR3C2 knockdown. *P < 0.05; **B** Transwell migration experiment validating that CQ increases the decrease in cells migration caused by NR3C2 overexpression. Upper: HCT116, lower: RKO, *P < 0.05(HCT116), #*P* < 0.05(RKO); **C** Transwell migration experiment validating that RAPA decreases the increase in cells migration caused by NR3C2 knockdown. Upper: SW620, lower: SW480, *P < 0.05(SW620), #*P* < 0.05(SW480); **D** Western blot detection of the impact of CQ on EMT-related proteins after NR3C2 overexpression in HCT116 cells. *P < 0.05 vs. vector, #*P* < 0.05 vs. NR3C2 + DMSO; **E** Western blot detection of the impact of CQ on EMT-related proteins after NR3C2 overexpression in RKO cells. *P < 0.05 vs. vector, #*P* < 0.05 vs. NR3C2 + DMSO; **F** Western blot detection of the impact of RAPA on EMT-related proteins after NR3C2 knockdown in SW620 cells. *P < 0.05 vs. Sh502, #*P* < 0.05 vs. ShD + DMSO; **G** Western blot detection of the impact of RAPA on EMT-related proteins after NR3C2 knockdown in SW480 cells. *P < 0.05 vs. Sh502, #*P* < 0.05 vs. ShD + DMSO.

we found that NR3C2 overexpression is associated with autophagy through the pathway enrichment of differential genes in RNA-seq (Fig. S4). we explored the connection between NR3C2 and autophagy by western blot. As NR3C2 expression increased, there was an elevation in LC3B expression and Beclin1 expression, a decrease in SQSTM1/p62 expression; Conversely, when NR3C2 expression was reduced, LC3B expression and beclin1 expression decreased, SQSTM1/p62 increased (Fig. 4A). NR3C2 overexpression promoted autophagy in CRC cells, while NR3C2 knockdown inhibited autophagy. The migration of HCT116 cells was suppressed when NR3C2 overexpression, but treatment with chloroquine (CQ) increased cells migration. Results in RKO cells were consistent with those in HCT116 cells (Fig. 4B). Conversely, NR3C2 knockdown increased the migration of SW620 cells, but treatment with rapamycin (RAPA) decreased cells migration. Similar results were observed in SW480 cells (Fig. 4C). Similarly, when NR3C2 is overexpressed, EMT-related proteins and autophagy related proteins are restored by the addition of CQ. These changes were statistically significant (Fig. 4D, E). On the other hand, when NR3C2 was knocked down, EMT-related proteins and autophagy related proteins are restored by the addition of RAPA. These changes were statistically significant (Fig. 4F, G). This further confirms that NR3C2 promotes autophagy and regulates the EMT process in CRC cells.

### NR3C2-SIRT1 axis regulates CRC cells autophagy

**Fig. 5:**
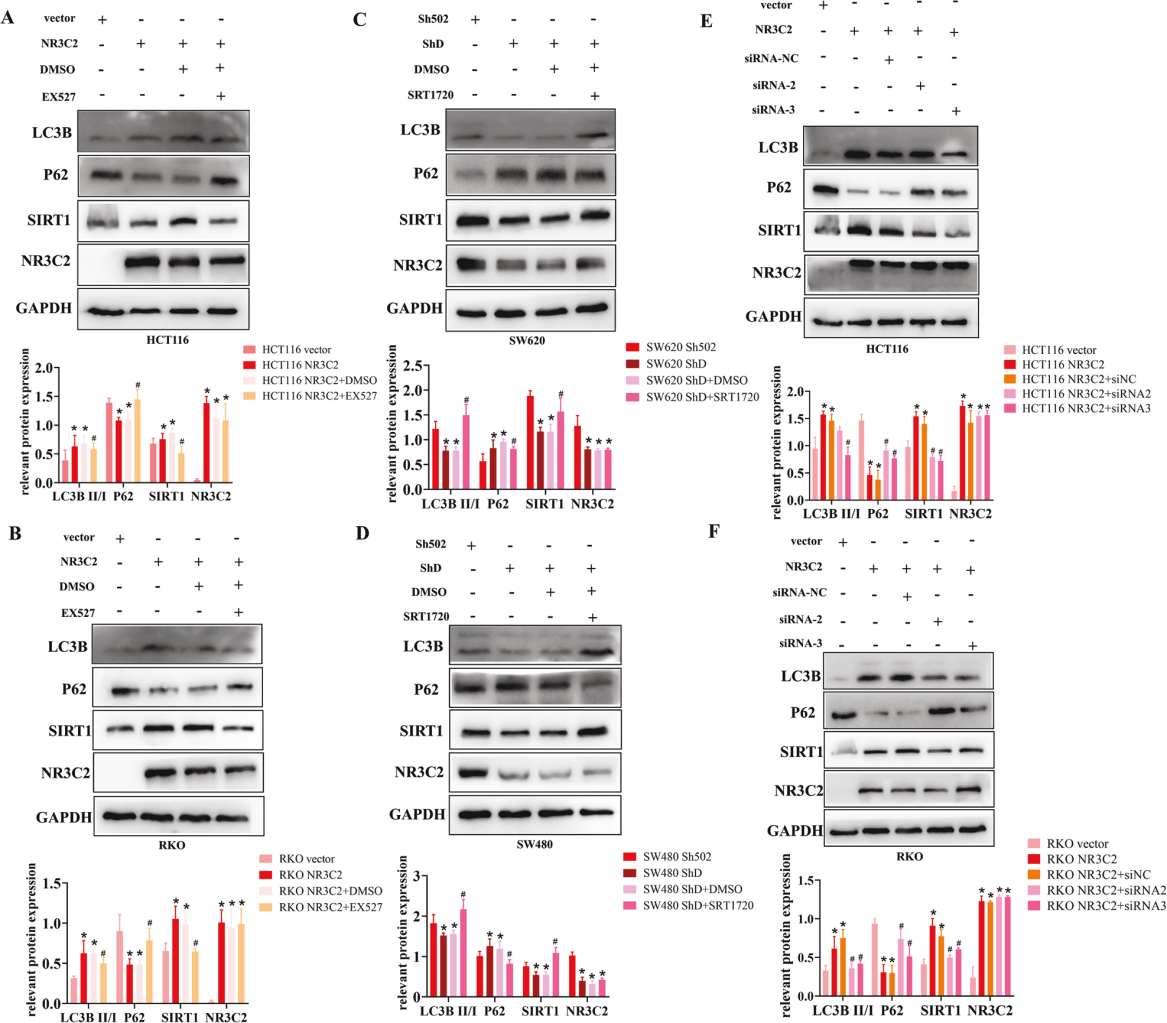
NR3C2-SIRT1 axis regulates CRC cells autophagy. **A** Western blot detection of changes in autophagy-related proteins after NR3C2 overexpression and addition of EX527 in HCT116 cells, Bottom: statistical chart, *P < 0.05 vs. vector, #*P* < 0.05 vs. NR3C2 + DMSO; **B** Western blot detection of changes in autophagy-related proteins after NR3C2 overexpression and addition of EX527 in RKO cells. Bottom: statistical chart, *P < 0.05 vs. vector, #*P* < 0.05 vs. NR3C2 + DMSO; **C** Western blot detection of changes in autophagy-related proteins after NR3C2 knockdown and addition of SRT1720 in SW620 cells. Bottom: statistical chart, *P < 0.05 vs. Sh502, #*P* < 0.05 vs. ShD + DMSO; **D** Western blot detection of changes in autophagy-related proteins after NR3C2 knockdown and addition of SRT1720 in SW480 cells. Bottom: statistical chart, *P < 0.05 vs. Sh502, #*P* < 0.05 vs. ShD + DMSO; **E** Western blot detection of changes in autophagy-related proteins after NR3C2 overexpression and addition of siSIRT1 in HCT116 cells; Bottom: statistical chart, *P < 0.05 vs. vector, #*P* < 0.05 vs. NR3C2 + siNC; **F** Western blot detection of changes in autophagy-related proteins after NR3C2 overexpression and addition of siSIRT1 in RKO cells; Bottom: statistical chart, *P < 0.05 vs. vector, #*P* < 0.05 vs. NR3C2 + siNC.

Therefore, combined with the experimental results, we investigated whether NR3C2 can modulate autophagy through SIRT1. As shown in Fig. 5A, NR3C2 overexpression led to a decrease in SQSTM1/p62 expression and an increase in LC3B expression. However, upon addition of EX527, SQSTM1/p62 expression increased, and LC3B expression decreased. Consistent results were observed in RKO cells, and these changes were statistically significant (Fig. 5B). Conversely, NR3C2 knockdown increased SQSTM1/p62 expression and decreased LC3B expression. However, upon addition of SRT1720, SQSTM1/p62 expression decreased, and LC3B expression increased, with statistical significance (Fig. 5C, D). Similarly, siRNA was also used to detect autophagy related proteins. When NR3C2 was overexpressed, the expression of P62 was decreased, while the expression of LC3B was increased; when siSIRT1 was added, the expression of P62 was increased again, and the expression of LC3B was decreased. The results of RKO cell experiments were consistent, which had statistical significance (Fig. 5E, F). This substantiates that NR3C2-SIRT1 axis regulates autophagy.

### NR3C2-SIRT1 axis suppresses lung metastasis in vivo

**Fig. 6:**
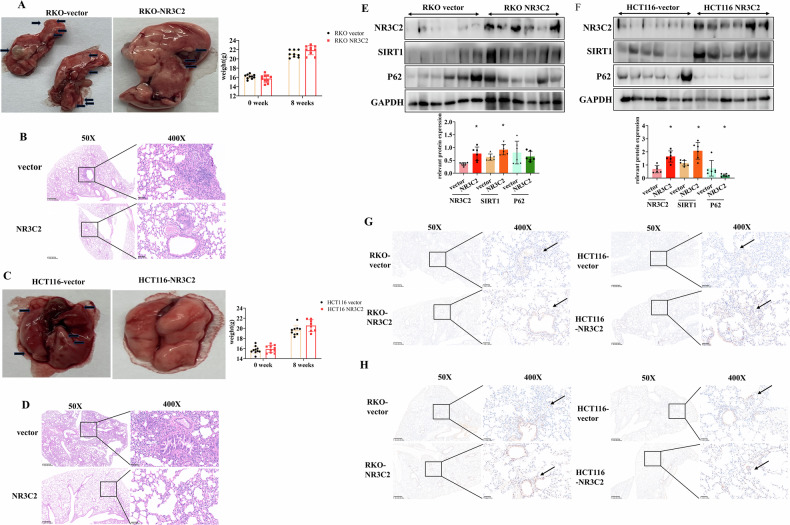
NR3C2-SIRT1 axis suppresses lung metastasis in vivo. **A** Photograph of mouse lung metastasis model using RKO cells, right: weight change of mice; **B** HE staining of mouse lung tissues for RKO-vector and RKO-NR3C2; **C** Photograph of mouse lung metastasis model using HCT116 cells, right: weight change of mice; **D** HE staining of mouse lung tissues for HCT116-vector and HCT116-NR3C2; **E** Western blot detection of NR3C2, SIRT1, and P62 expression in the lung tissues of mice in the RKO group, *P < 0.05; **F** Western blot detection of NR3C2, SIRT1, and P62 expression in the lung tissues of mice in the HCT116 group, *P < 0.05; **G** IHC detection of NR3C2 expression in mouse lung tissues (50X and 400X); **H** IHC detection of SIRT1 expression in mouse lung tissues(50X and 400X).

**Fig. 7:**
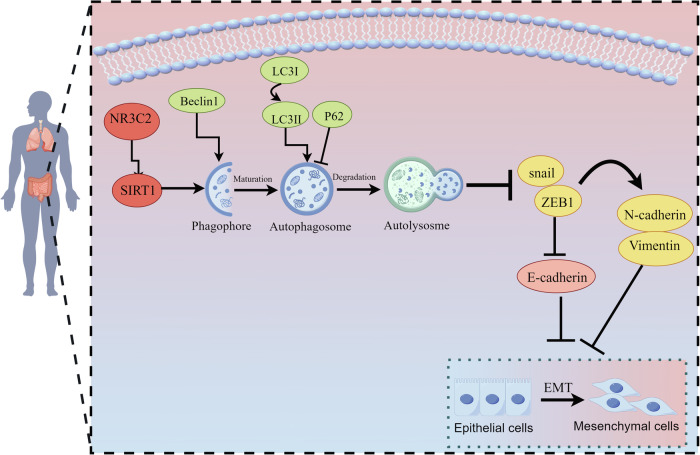
Graphical model for the mechanism (By Figdraw). NR3C2 and SIRT1 constitute a pivotal signaling axis that modulates EMT in CRC cells through the intricate mechanism of autophagy.

We established a lung metastasis model. Macroscopically, there were no visible differences between the two groups. After 8 weeks, both groups of mice exhibited an increase in body weight, with RKO-vector mice showing a smaller weight gain compared to RKO-NR3C2 mice. In the RKO-vector group, tumor cells migrated to lung tissues, forming tumors of varying sizes, while the RKO-NR3C2 group exhibited a significant reduction in metastatic tumors (Figs. S5 and 6A). HE staining analysis at 400X magnification revealed abundant tumor cells clusters in the lung tissues of the RKO-vector group, whereas the RKO-NR3C2 group displayed fewer tumor cells clusters (Fig. 6B). Similarly, in the HCT116-NR3C2 group compared to the HCT116-vector group, there was a reduction in the number of tumor metastases, accompanied by a greater increase in body weight (Figs. S6 and 6C). HE results at 400X magnification clearly showed tumor cell clusters in the lung tissues of the HCT116-vector group, whereas no tumor metastasis was observed in the HCT116-NR3C2 group (Fig. 6D). Subsequently, we conducted Western blot analysis on mouse lung tissues, revealing higher expression levels of NR3C2 and SIRT1, along with lower SQSTM1/p62 expression in the RKO-NR3C2 and HCT116-NR3C2 groups, consistent with in vitro results (Fig. 6E, F). IHC staining also confirmed higher expression of NR3C2 and SIRT1 in the RKO-NR3C2 and HCT116-NR3C2 groups (Fig. 6G, H). This section, through the construction of a metastasis model, validates that NR3C2 - SIRT1 axis promotes autophagy and inhibits lung metastasis in vivo (Fig. 7).

### NR3C2 was downregulation in the CRC tissues

Finally, we examined whether NR3C2 expression may be correlated with CRC progression. We collected specimens from 246 CRC patients. Through bioinformatics analysis, we observed that the expression level of NR3C2 is significantly lower in tumor tissues compared to normal tissues. Additionally, as the N stage of tumors increases, the expression of NR3C2 gradually decreases. This trend is consistent in both COAD and READ (Fig. S7A, B). The analysis of NR3C2 expression in samples from enrolled patients, revealed that it in tumor tissues was lower than that in paired adjacent non-cancerous tissues by IHC. Moreover, it demonstrates a decreasing trend correlated with the rising N stage (Fig. S7C). The Western blot analysis of 32 randomly selected samples indicated a noteworthy reduction in the protein expression of NR3C2 in tumor tissues compared to adjacent non-cancerous tissues (Fig. S7D). qRT-PCR analysis of all enrolled patient samples (246 in total), we demonstrated that the RNA expression of NR3C2 in tumor tissues is markedly lower than in adjacent non-cancerous tissues. Notably, NR3C2 expression is unrelated to age and gender. Particularly significant is the observation that with higher N and M stages, the expression of NR3C2 shows a gradual decrease ((Fig. S7E). These results confirm the downregulation of NR3C2 in tumors and its negative correlation with tumor staging.

### SIRT1 was downregulation in the CRC tissues

Since NR3C2 regulates SIRT1, we also examined the expression of SIRT1 in CRC. Initially, through TCGA database analysis, we assessed the pan-cancer expression of SIRT1 and observed its low expression across various tumors such as BLCA, BRCA, CESC, KICH, KIRP, COAD and READ ((Fig. S8A). Focusing on COAD and READ, SIRT1 expression was consistently lower in tumors compared to normal tissues, although no clear trend of decreasing expression with higher tumor staging was observed ((Fig. S8B–D). To validate these findings, 16 randomly selected clinical samples were detected by Western blot, confirming the low expression of SIRT1 in tumors and its higher expression in adjacent non-cancerous tissues (Fig. S8E). Subsequently, 60 samples were chosen for qRT-PCR analysis, reaffirming that SIRT1 mRNA levels in tumors were lower than in adjacent non-cancerous tissues. Importantly, SIRT1 expression showed no correlation with age or gender, but notably decreased with higher N staging ((Fig. S8F). This substantiates the role of SIRT1 in CRC progression.

## Discussion

CRC ranks as the second most common malignant tumor and the second leading cause of cancer-related deaths worldwide [30]. With the continuous progress in developing countries, it is projected that the global incidence of new CRC cases will increase by nearly 2.5 million by 2035 [31]. Despite significant advancements in medical technology and multidisciplinary comprehensive treatments for mCRC in recent years, the high mortality rate associated with mCRC has not shown significant improvement. The 5-year survival rate for early-stage CRC exceeds 71%, while the 5-year survival rate for mCRC drops to 14% [31].

NR3C2 exhibits widespread expression across various tissues and cell types, including the gastrointestinal tract, immune cells, brain, heart, bones. In polarized epithelial cells, its primary target is the ligand aldosterone [32], NR3C2 is also involved in the development of various tumor cells, including pancreatic ductal adenocarcinoma [12], hepatocellular carcinoma [14, 15], and breast cancer [18, 19]. However, research on the regulation of downstream molecules by NR3C2 is currently scarce. Through RNA-seq, we have identified a downstream molecule, SIRT1, which represents a new finding in the study of NR3C2. SIRT1, as the most tumor-associated subtype of the sirtuins family, is a regulator of autophagy [33].

SIRT1 has a controversial point in the current study, we believe SIRT1 plays intricate roles in the tumorigenesis process, acting as both a tumor suppressor by inhibiting transcription to prevent tumor initiation and as a promoter of EMT, thereby inducing tumor metastasis and progression. In the realm of CRC research, there exist conflicting reports on the role of SIRT1, with some studies suggesting its pro-carcinogenic effects [34]. In a meta-analysis incorporating seven studies, SIRT1 was found to be upregulated more frequently in lymph node metastasis and stages T3/T4 in CRC patients. Its overexpression typically signifies a poorer prognosis in CRC patients [35]. Feifei Cheng [36] provided mechanistic insight into the regulation of Fra-1 by SIRT1, SIRT1-Fra-1 axis directly regulated the EMT process of tumors. At the same time, the high expression of SIRT1 predicted the high expression of Fra-1 in CRC patients, and the metastasis was increased, which was positively correlated with OS. These collective findings suggest the pro-carcinogenic role of SIRT1 and propose it as a potential biomarker for monitoring CRC progression. Conversely, studies have indicated that SIRT1 overexpression serves as a positive prognostic factor for CRC, indicate its tumor inhibitory functions [37]. SIRT1 exerts anti-cancer effects in CRC by suppressing oncogenes such as β-catenin and survivin. Downregulated in CRC, SIRT1’s expression gradually decreases with tumor progression, showing a strong correlation with overall survival in CRC patients [38]. A report suggests that SIRT1 suppresses CRC metastasis in vitro and in vivo as a negative regulator for miR-15b-5p transcription [39].

The contradictory roles of SIRT1 in cancer, involving both the promotion of EMT, inhibition of tumor immunity, and maintenance of tumor stem cells characteristics, as well as its tumor-suppressive effects through the inhibition of oncogene expression and CRC cells metabolism, highlight the dual nature of SIRT1 in CRC. Combining these findings with previous studies, it is discovered that NR3C2 inhibits CRC proliferation by suppressing glucose metabolism [11]. Token together, NR3C2, as the upstream of SIRT1, directly forms a signal axis with SIRT1 to regulate the autophagy and EMT processes of CRC. In summary, we believe that the dual function of SIRT1 in CRC is due to its regulation by different upstream molecules in different stages of CRC, as well as the main target pathways in the occurrence and progression of CRC, and more importantly, the influence of tumor microenvironment.

In previous studies, we confirmed that NR3C2 regulates AMPK, which acts as a conserved energy sensor, is the target of SIRT1 for regulation of cellular metabolism. SIRT1 is a selective substrate for autophagy during cell aging and plays a central role in autophagy regulation. Autophagy influences tumor development by suppressing tumor growth, altering the tumor microenvironment, while paradoxically promoting tumor cell survival [40]. AMPK promotes autophagy by phosphorylating BECN1 at Thr388 [41]. The activation of AMPK-dependent autophagy exerts anti-tumor effects in the early stages of tumor formation. Mice with liver-specific Atg7 knockout develop liver tumors, while knockout of SQSTM1/p62 reduces tumor size. These findings suggest that sustained autophagy can inhibit liver tumors, whereas SQSTM1/p62 accumulation favors tumorigenesis [42].

NR3C2, as an upstream regulator of SIRT1, directly forms a signaling axis with SIRT1, jointly regulating the autophagy and EMT processes in CRC. In this study, inhibitors and inducers of SIRT1, inhibitors and inducers of autophagy have also been used to verify that autophagy activation can regulate EMT by fine-tuning various NR3C2-SIRT1 signaling axis, either inhibiting or enhancing the process. On one hand, autophagy activation provides energy and essential nutrients for EMT during the metastatic cascade, aiding cell survival in stressful environments and intracellular conditions. Autophagy can restrict the EMT phenotype by increasing the instability of key EMT proteins. For example, activated autophagy induces the acetylation and destabilization of the transcription factor Snail [43]. In contrast, autophagy deficiency stabilizes the TWIST1 protein through the accumulation of SQSTM1/p62 [44]. Similarly, certain chemokines in the tumor microenvironment also regulate EMT via autophagy. CCL2 promotes the metastasis and EMT of non-small cell lung cancer through the PI3K/AKT/mTOR axis and autophagy signaling pathways [45]. On the other hand, autophagy exhibits anti-cancer functions, selectively downregulating key transcription factors of EMT in the early stages, thereby impeding metastasis. Beclin1 is the homologous gene of the yeast gene ATG6/VPS30. It binds with VPS34 to form a complex that can induce autophagy. It inhibits the EMT process by downregulating the expression of ZEB1, WNT1 and NF-κB [46]. Similarly, the inhibition of BEC1 and ATG7 in cells reduces epithelial markers such as E-cadherin and increases the expression levels of mesenchymal markers such as vimentin, MMP9, and Snail. When Beclin-1 is knocked out, tumor cells acquire EMT mesenchymal characteristics by stabilizing the mRNA level of ZEB1 [43]. Therefore, we believe that autophagy and EMT play indispensable roles in the occurrence and development of tumors. There is an urgent need to discover new molecules that regulate these two processes, which will help further understand the mechanism of EMT and obtain effective anticancer treatment strategies by blocking metastasis.

## Conclusion

NR3C2 as an anti - oncogene and is downregulated in CRC tissues. NR3C2 overexpression / knockdown affects the migratory capacity and EMT of CRC cells. Mechanistically, NR3C2 forms a signaling axis with its downstream regulatory molecule SIRT1, modulating EMT in CRC cells via autophagy. Both NR3C2 and SIRT1 serve as key regulatory factors in CRC and are intimately associated with the development and progression of CRC. Therefore, the NR3C2-SIRT1 signaling axis represents an important pathway for inhibiting the metastasis of CRC, and provides a potential therapeutic target for CRC treatment.

## Supplementary information

The NR3C2-SIRT1 signaling axis promotes autophagy and inhibits epithelial mesenchymal transition in colorectal cancer

uncropped WB blots

## Data Availability

All data generated or analyzed during this study are included in this article.
