# Supplementary material for: The NR3C2-SIRT1 signaling axis promotes autophagy and inhibits epithelial mesenchymal transition in colorectal cancer
Source: Cell Death Dis. 2025 Apr 14;16(1):295. doi: 10.1038/s41419-025-07575-3 (PMC11997134; doi:10.1038/s41419-025-07575-3)
Supplement: Supplementary file 2 — uncropped WB blots [file 41419_2025_7575_MOESM2_ESM.pdf]

Fig 1 I HCT116 cells

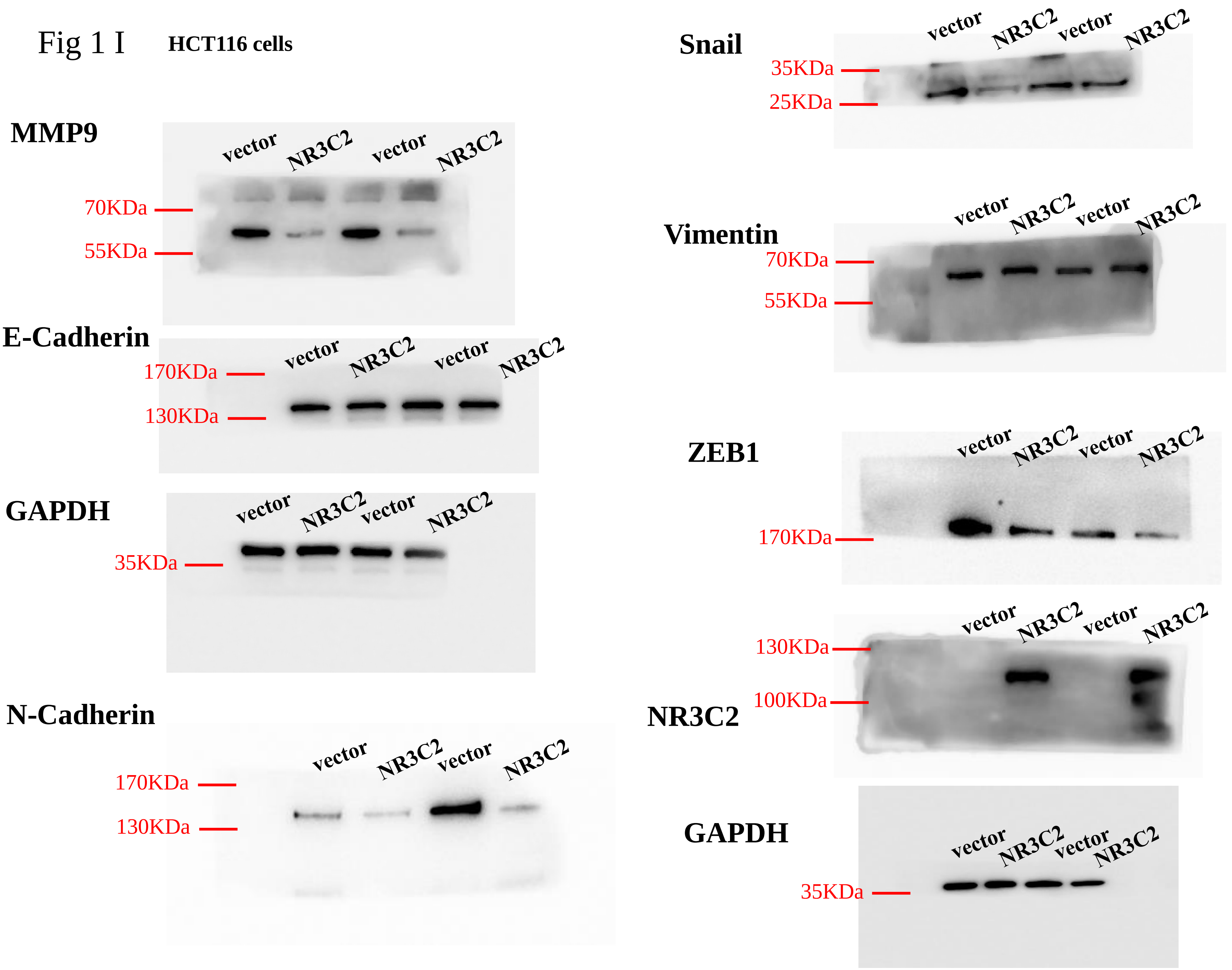

Fig 1 J

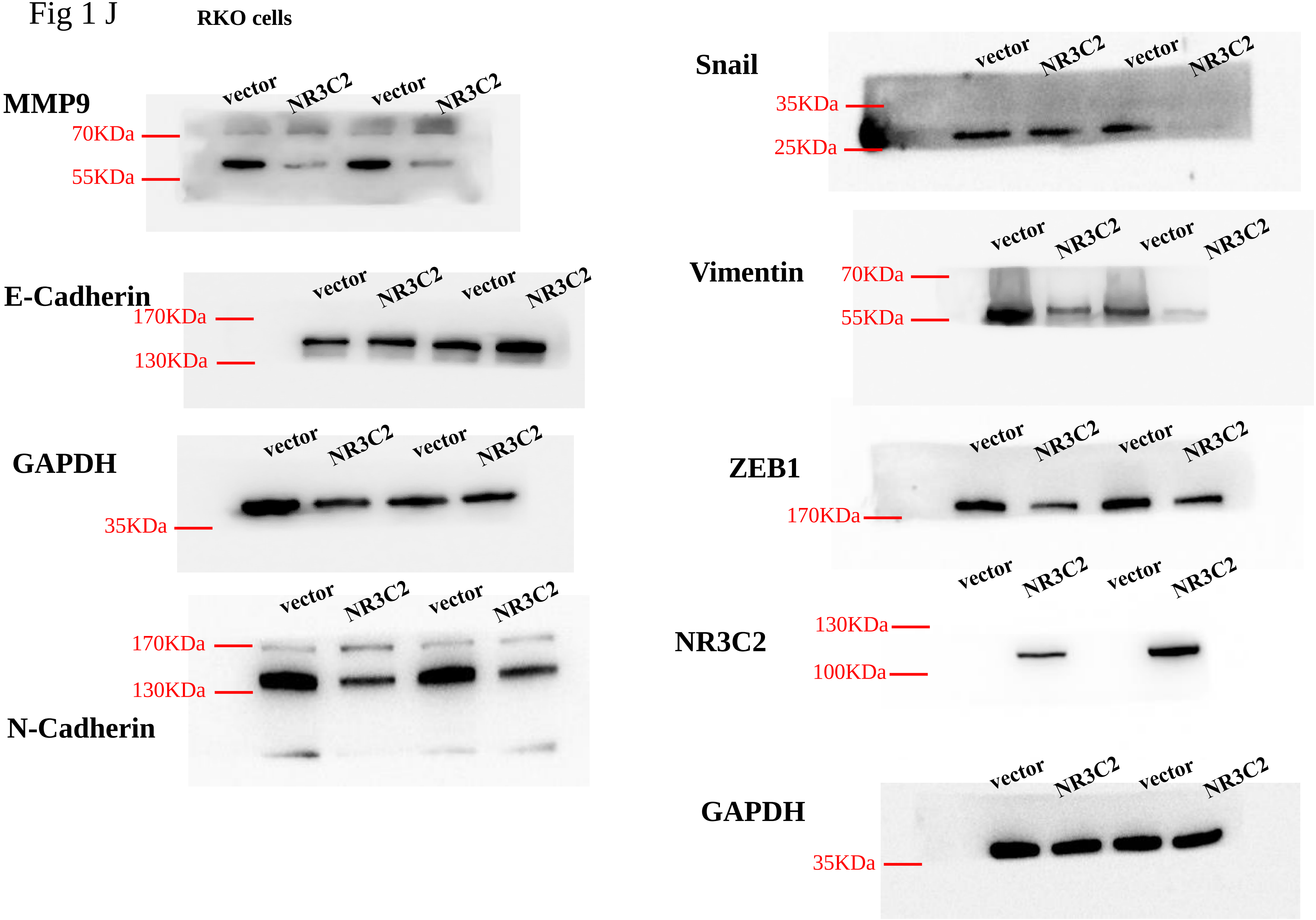
RKO cells
MMP9
vector
NR3C2
vector
NR3C2
70KDa
55KDa
E-Cadherin
vector
NR3C2
vector
NR3C2
170KDa
130KDa
GAPDH
vector
NR3C2
vector
NR3C2
35KDa
N-Cadherin
vector
NR3C2
vector
NR3C2
170KDa
130KDa
Snail
vector
NR3C2
vector
NR3C2
35KDa
25KDa
Vimentin
vector
NR3C2
vector
NR3C2
70KDa
55KDa
ZEB1
vector
NR3C2
vector
NR3C2
170KDa
NR3C2
vector
NR3C2
vector
NR3C2
130KDa
100KDa
GAPDH
vector
NR3C2
vector
NR3C2
35KDa

Fig 1 K SW620 cells

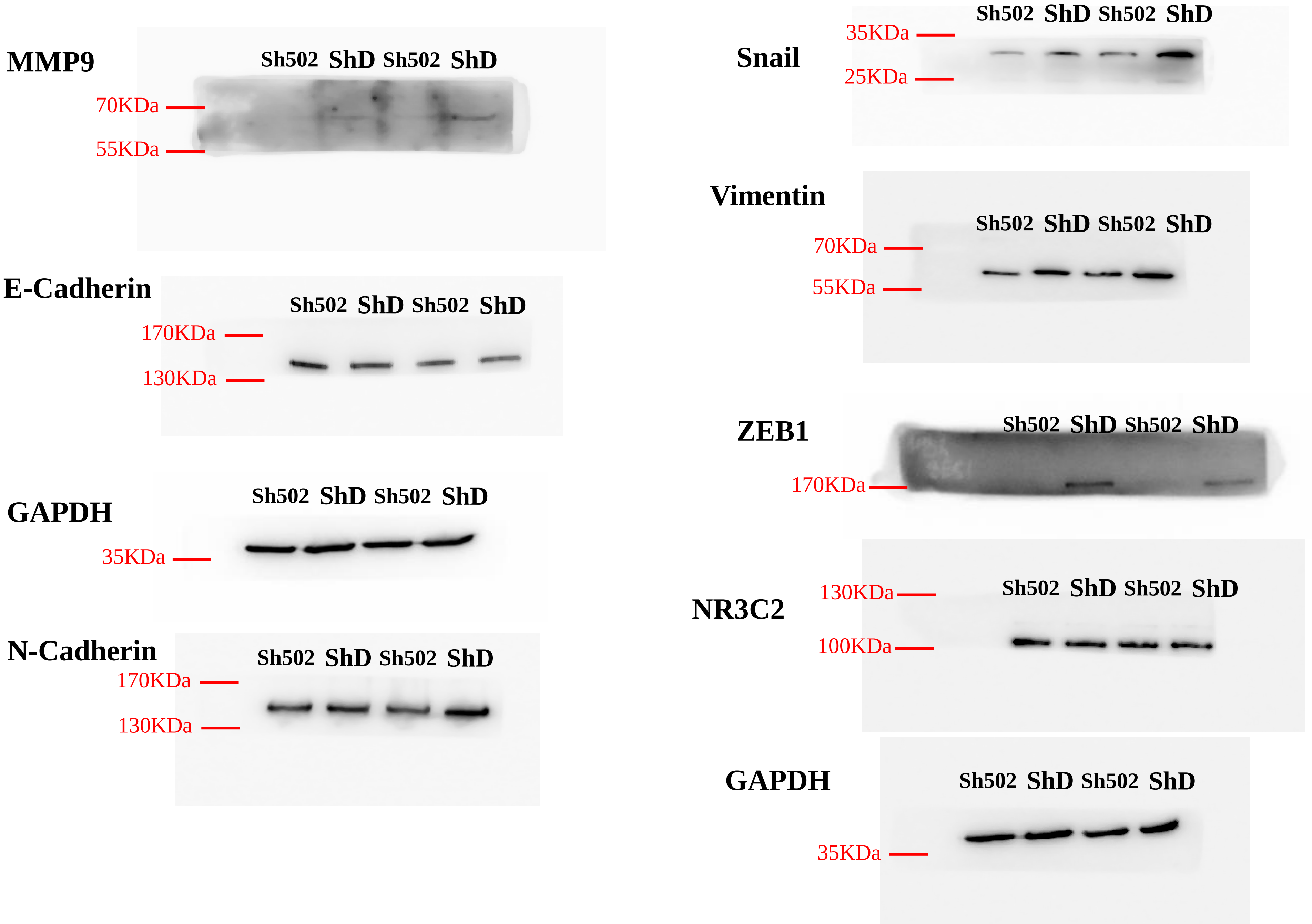

Fig 1 L

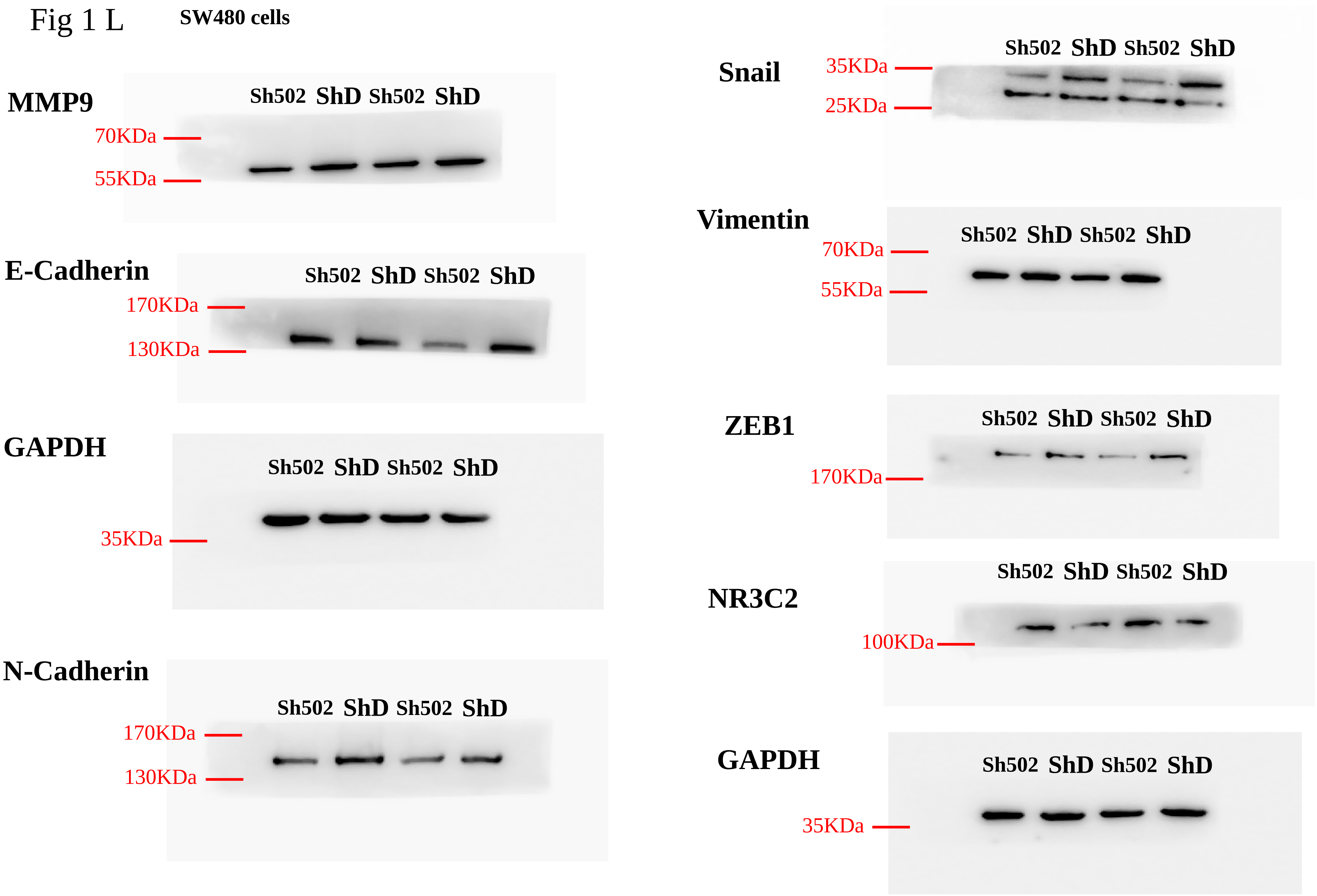
SW480 cells
MMP9
Sh502 ShD Sh502 ShD
70KDa
55KDa
E-Cadherin
Sh502 ShD Sh502 ShD
170KDa
130KDa
GAPDH
Sh502 ShD Sh502 ShD
35KDa
N-Cadherin
Sh502 ShD Sh502 ShD
170KDa
130KDa
Snail
Sh502 ShD Sh502 ShD
35KDa
25KDa
Vimentin
Sh502 ShD Sh502 ShD
70KDa
55KDa
ZEB1
Sh502 ShD Sh502 ShD
170KDa
NR3C2
Sh502 ShD Sh502 ShD
100KDa
GAPDH
Sh502 ShD Sh502 ShD
35KDa

Fig 2 C

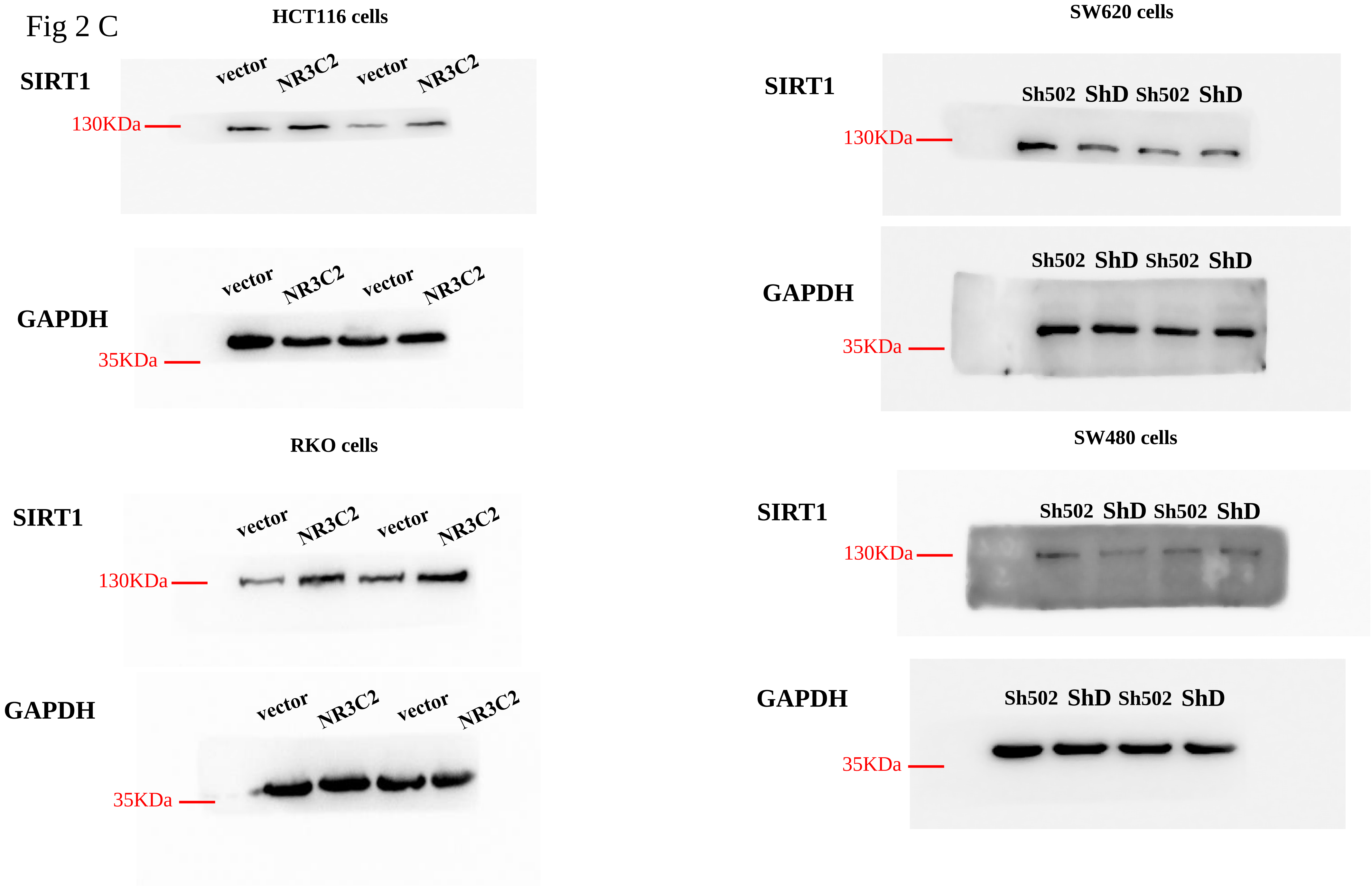
HCT116 cells
SIRT1
vector NR3C2 vector NR3C2
130KDa
GAPDH
vector NR3C2 vector NR3C2
35KDa
RKO cells
SIRT1
vector NR3C2 vector NR3C2
130KDa
GAPDH
vector NR3C2 vector NR3C2
35KDa
SW620 cells
SIRT1
Sh502 ShD Sh502 ShD
130KDa
GAPDH
Sh502 ShD Sh502 ShD
35KDa
SW480 cells
SIRT1
Sh502 ShD Sh502 ShD
130KDa
GAPDH
Sh502 ShD Sh502 ShD
35KDa

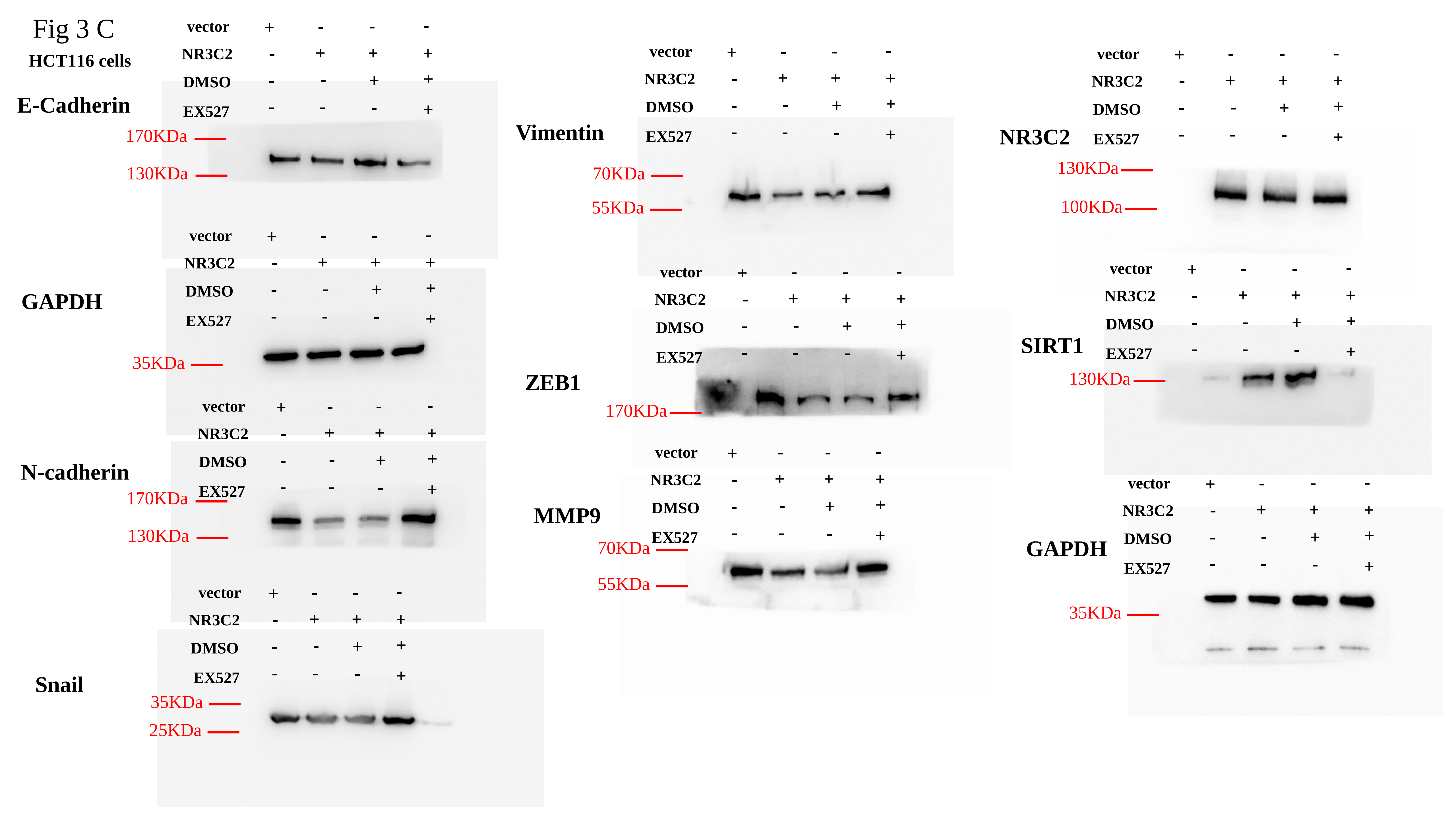
Fig 3 C
HCT116 cells
E-Cadherin
vector + - - -
NR3C2 - + + +
DMSO - - + +
EX527 - - - +
170KDa
130KDa
GAPDH
vector + - - -
NR3C2 - + + +
DMSO - - + +
EX527 - - - +
35KDa
N-cadherin
vector + - - -
NR3C2 - + + +
DMSO - - + +
EX527 - - - +
170KDa
130KDa
Snail
vector + - - -
NR3C2 - + + +
DMSO - - + +
EX527 - - - +
35KDa
25KDa
Vimentin
vector + - - -
NR3C2 - + + +
DMSO - - + +
EX527 - - - +
70KDa
55KDa
ZEB1
vector + - - -
NR3C2 - + + +
DMSO - - + +
EX527 - - - +
170KDa
MMP9
vector + - - -
NR3C2 - + + +
DMSO - - + +
EX527 - - - +
70KDa
55KDa
NR3C2
vector + - - -
NR3C2 - + + +
DMSO - - + +
EX527 - - - +
130KDa
100KDa
SIRT1
vector + - - -
NR3C2 - + + +
DMSO - - + +
EX527 - - - +
130KDa
GAPDH
vector + - - -
NR3C2 - + + +
DMSO - - + +
EX527 - - - +
35KDa

Fig 3 D

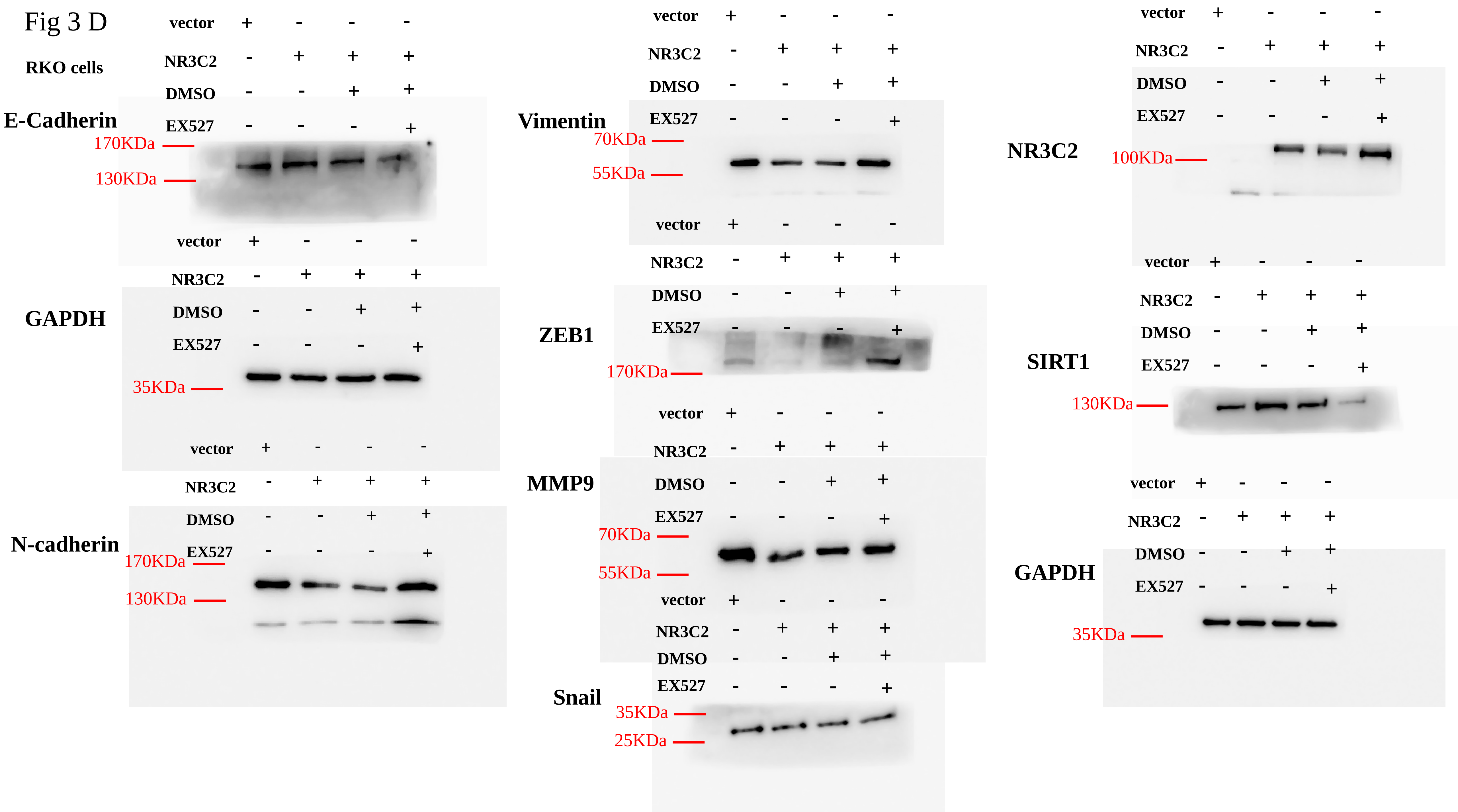
RKO cells
vector
NR3C2
DMSO
EX527
E-Cadherin
170KDa
130KDa
GAPDH
35KDa
N-cadherin
Vimentin
70KDa
55KDa
ZEB1
MMP9
Snail
25KDa
NR3C2
100KDa
SIRT1

Fig 3 E

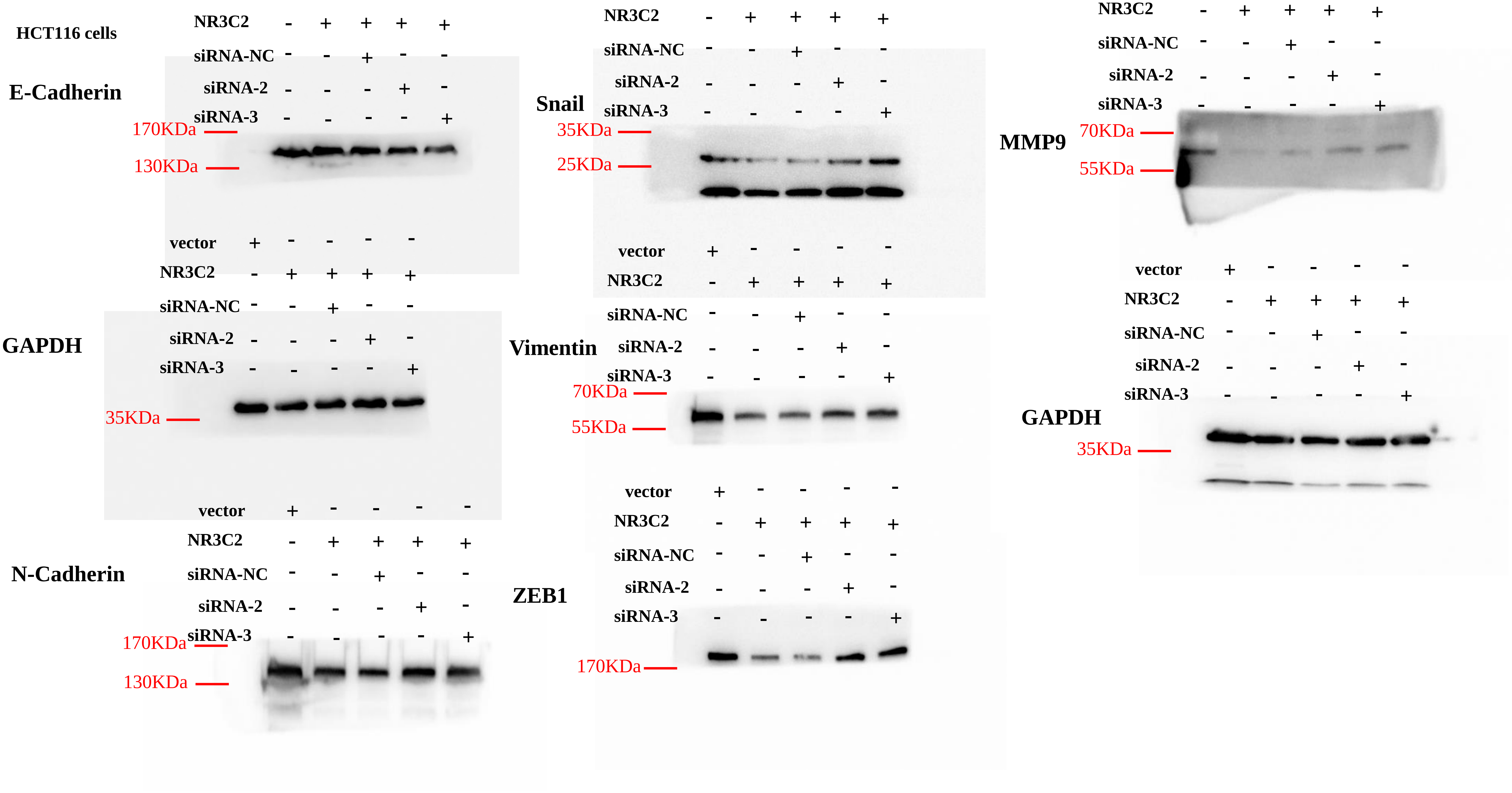
HCT116 cells
E-Cadherin
GAPDH
N-Cadherin
Snail
Vimentin
ZEB1
MMP9
vector
NR3C2
siRNA-NC
siRNA-2
siRNA-3
170KDa
130KDa
35KDa
25KDa
70KDa
55KDa

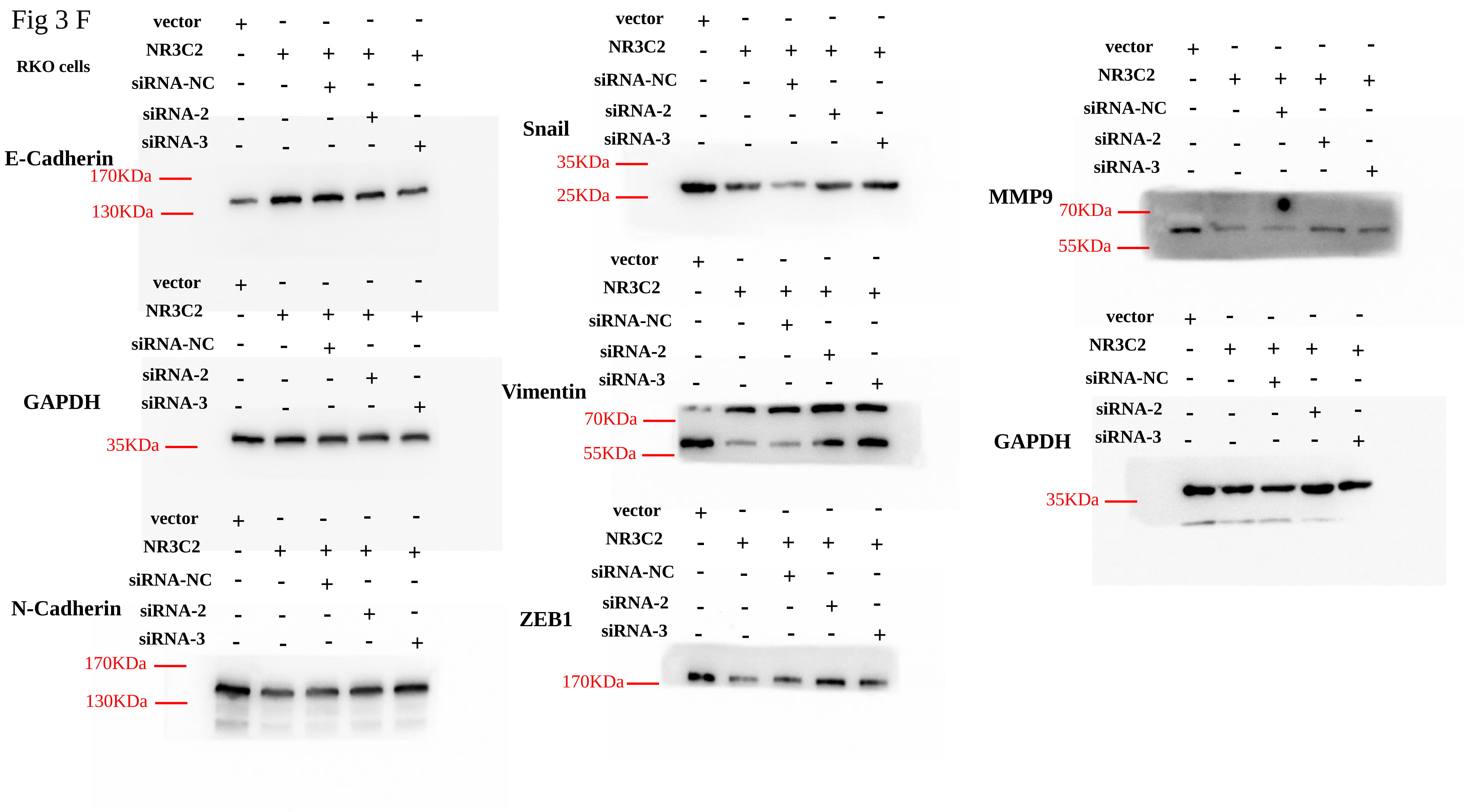
Fig 3 F
RKO cells
vector
NR3C2
siRNA-NC
siRNA-2
siRNA-3
E-Cadherin
170KDa
130KDa
GAPDH
35KDa
N-Cadherin
170KDa
130KDa
Snail
35KDa
25KDa
Vimentin
70KDa
55KDa
ZEB1
170KDa
MMP9
70KDa
55KDa
GAPDH
35KDa

Fig 3 G

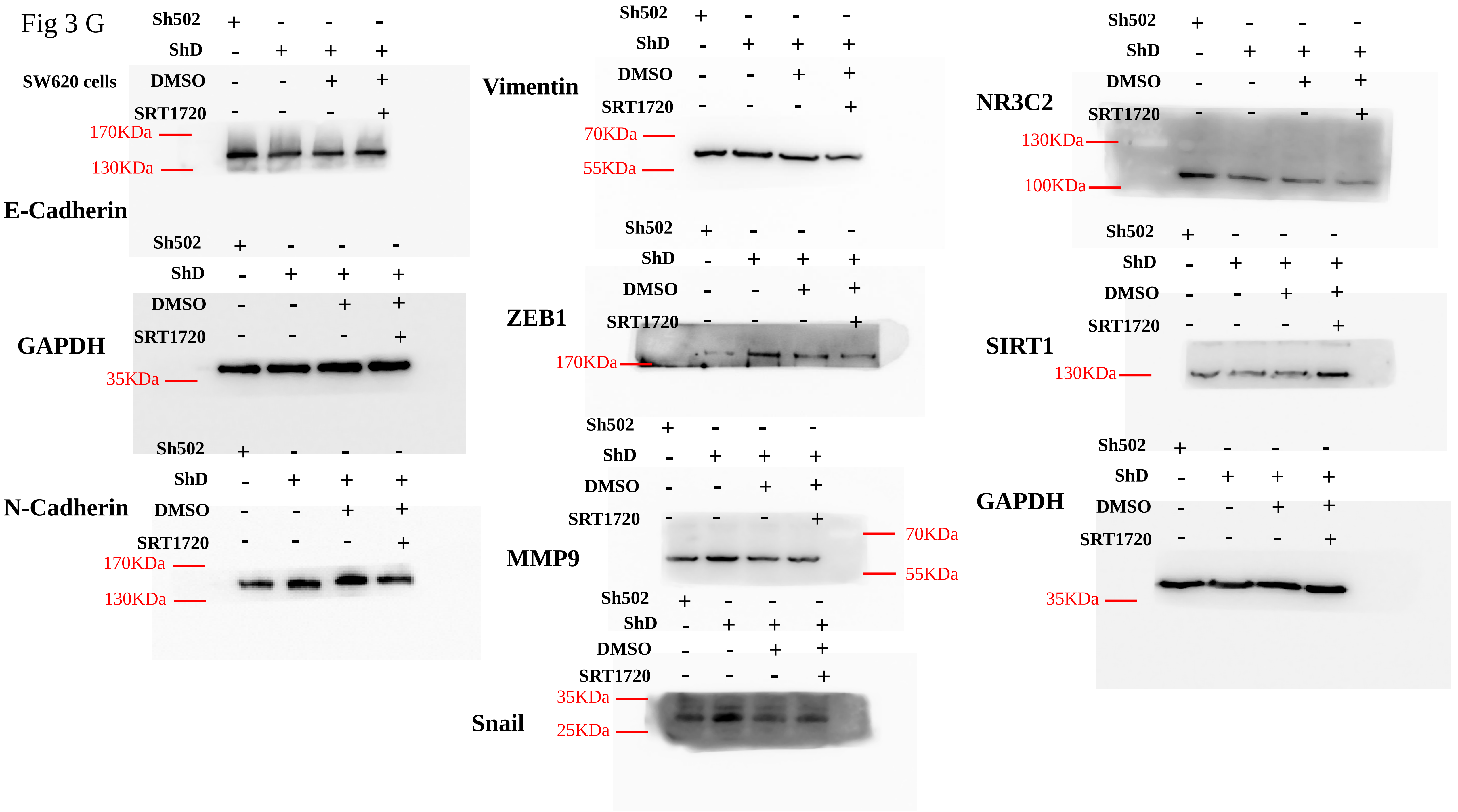
SW620 cells
Sh502 + - - -
ShD - + + +
DMSO - - + +
SRT1720 - - - +
170KDa
130KDa
E-Cadherin
GAPDH
35KDa
N-Cadherin
170KDa
130KDa
Vimentin
70KDa
55KDa
ZEB1
170KDa
MMP9
70KDa
55KDa
Snail
35KDa
25KDa
NR3C2
130KDa
100KDa
SIRT1
130KDa
GAPDH
35KDa

Fig 3 H

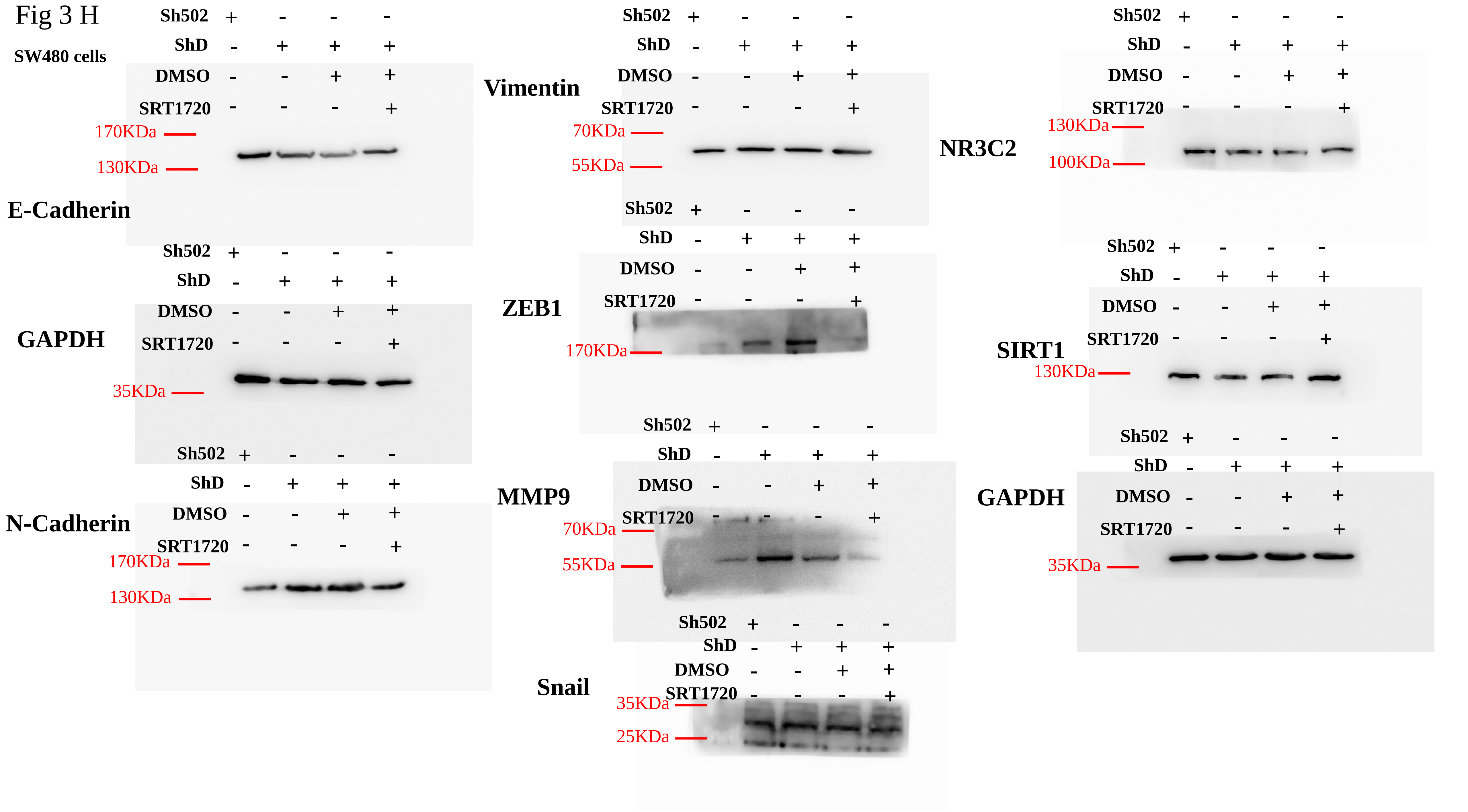
SW480 cells
Sh502 + - - -
ShD - + + +
DMSO - - + +
SRT1720 - - - +
E-Cadherin
170KDa
130KDa
GAPDH
35KDa
N-Cadherin
170KDa
130KDa
Vimentin
70KDa
55KDa
ZEB1
170KDa
MMP9
70KDa
55KDa
Snail
35KDa
25KDa
NR3C2
130KDa
100KDa
SIRT1
130KDa
GAPDH
35KDa

Fig 4 A

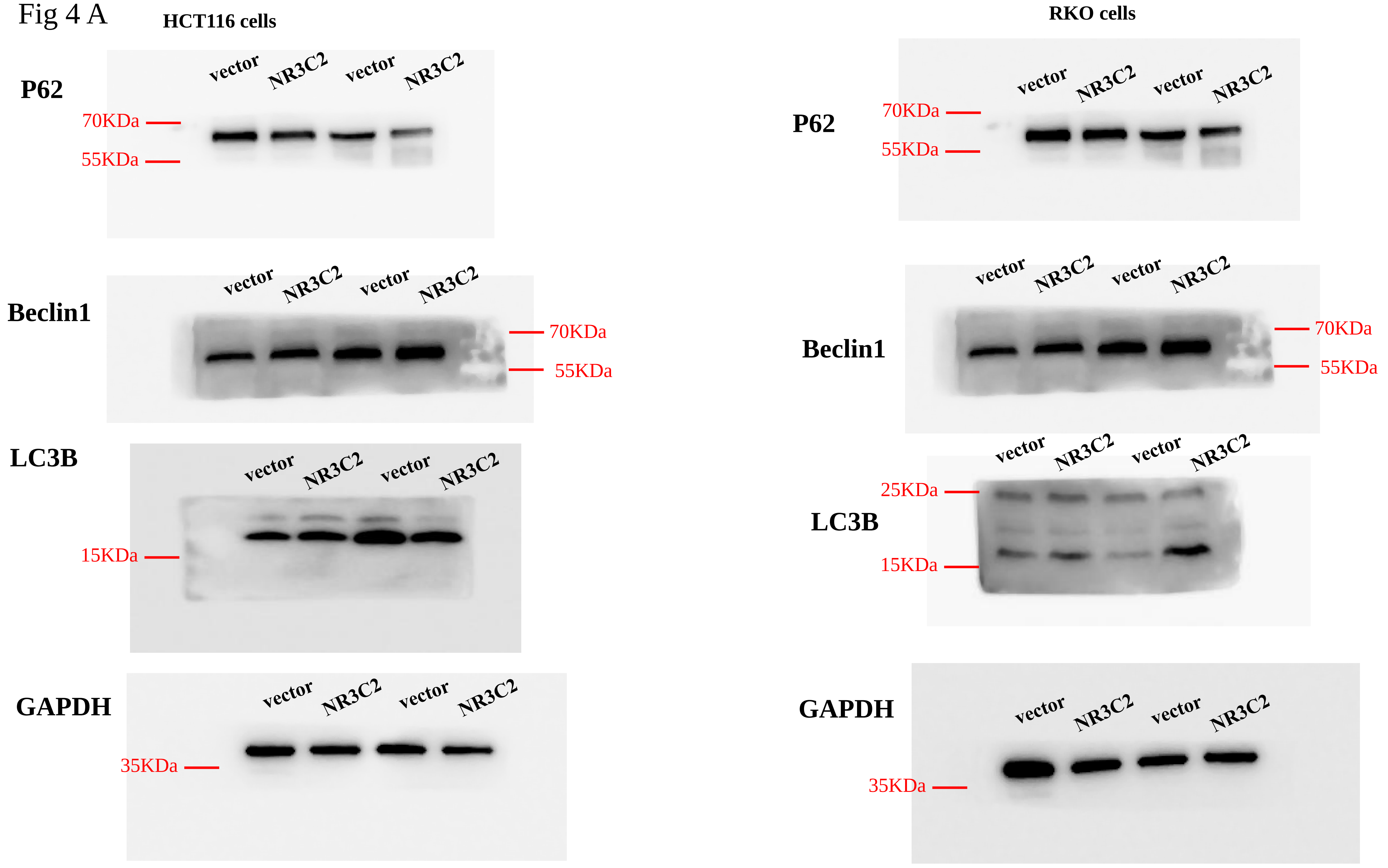

HCT116 cells
RKO cells
P62
Beclin1
LC3B
GAPDH
vector
NR3C2
vector
NR3C2
70KDa
55KDa
25KDa
15KDa
35KDa

Fig 4 A

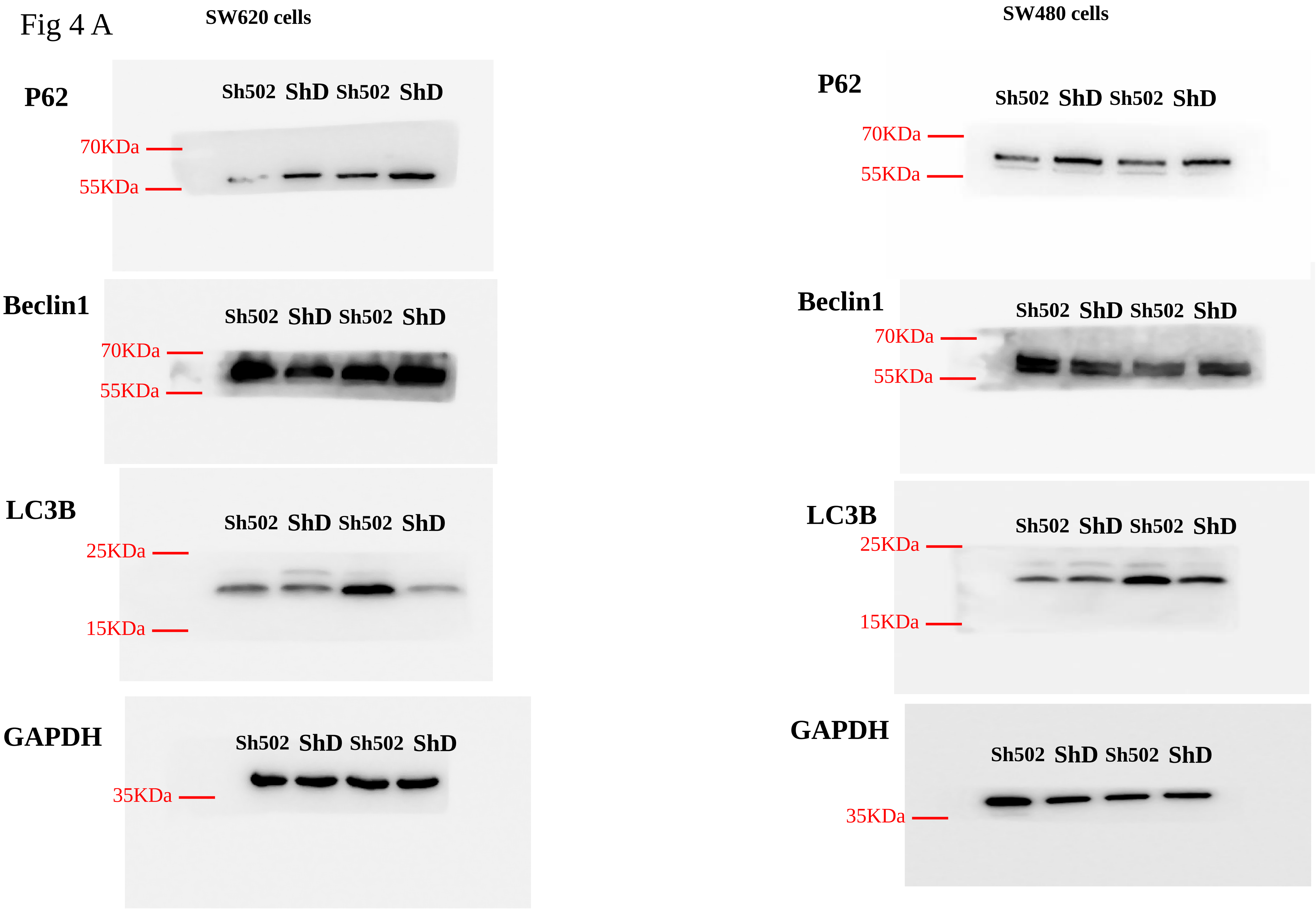
SW620 cells
SW480 cells
P62
Sh502 ShD Sh502 ShD
70KDa
55KDa
Beclin1
Sh502 ShD Sh502 ShD
70KDa
55KDa
LC3B
Sh502 ShD Sh502 ShD
25KDa
15KDa
GAPDH
Sh502 ShD Sh502 ShD
35KDa
P62
Sh502 ShD Sh502 ShD
70KDa
55KDa
Beclin1
Sh502 ShD Sh502 ShD
70KDa
55KDa
LC3B
Sh502 ShD Sh502 ShD
25KDa
15KDa
GAPDH
Sh502 ShD Sh502 ShD
35KDa

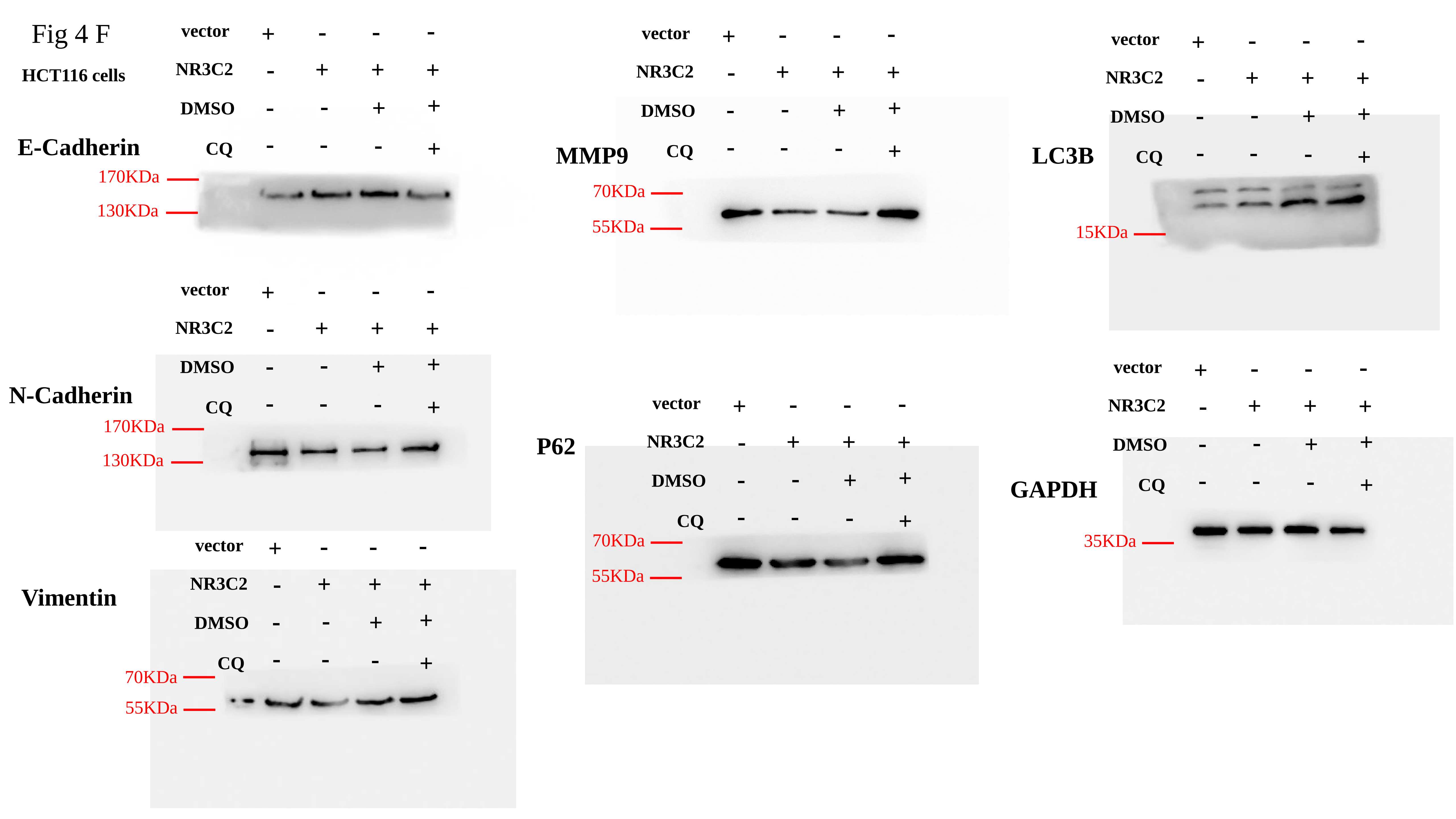

Fig 4 F
HCT116 cells
vector + - - -
NR3C2 - + + +
DMSO - - + +
CQ - - - +
E-Cadherin
170KDa
130KDa
MMP9
70KDa
55KDa
LC3B
15KDa
N-Cadherin
170KDa
130KDa
P62
70KDa
55KDa
GAPDH
35KDa
Vimentin
70KDa
55KDa

Fig 4 F

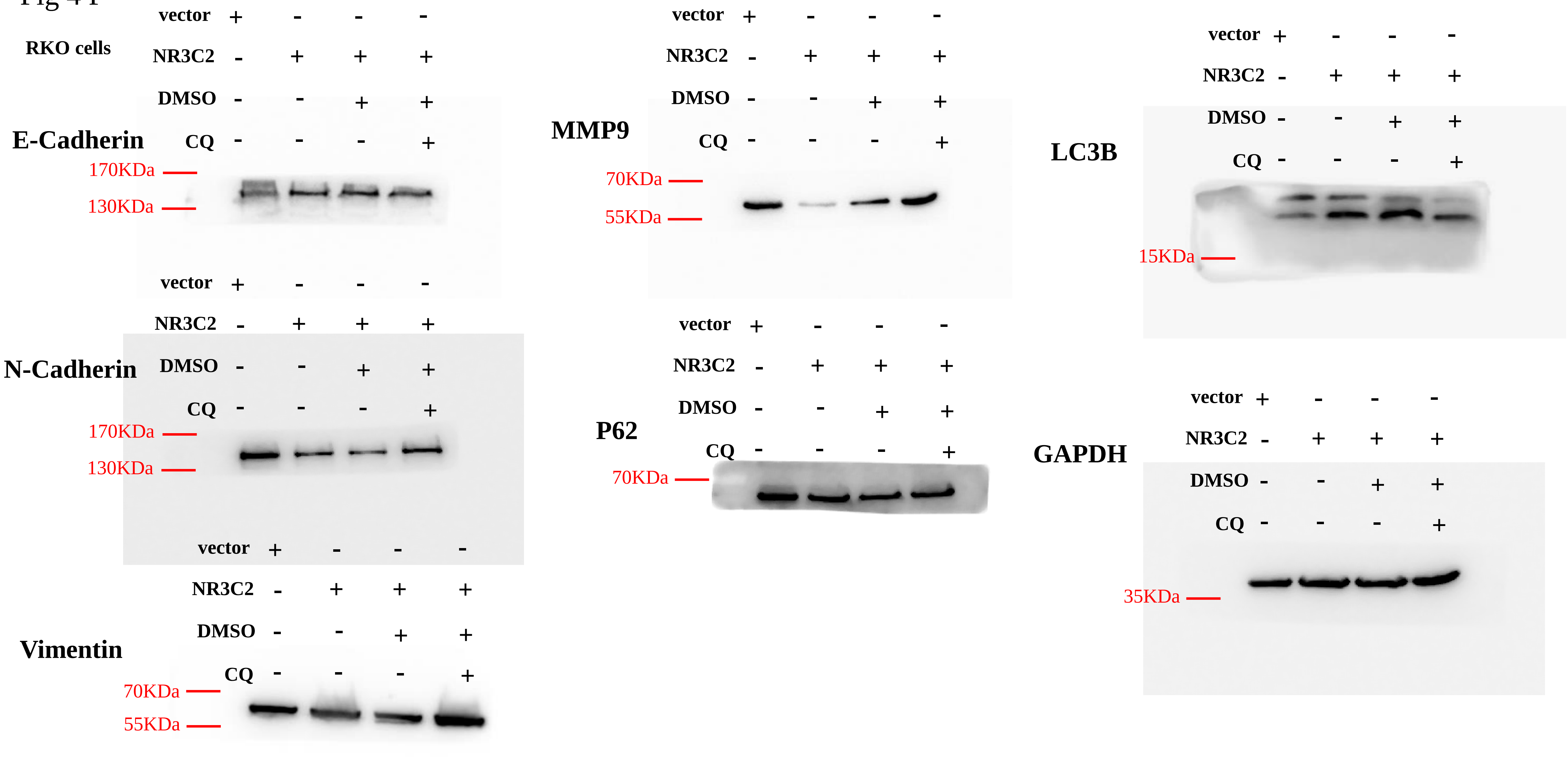
RKO cells
vector + - - -
NR3C2 - + + +
DMSO - - + +
CQ - - - +
E-Cadherin
170KDa
130KDa
N-Cadherin
170KDa
130KDa
Vimentin
70KDa
55KDa
MMP9
70KDa
55KDa
P62
70KDa
LC3B
15KDa
GAPDH
35KDa

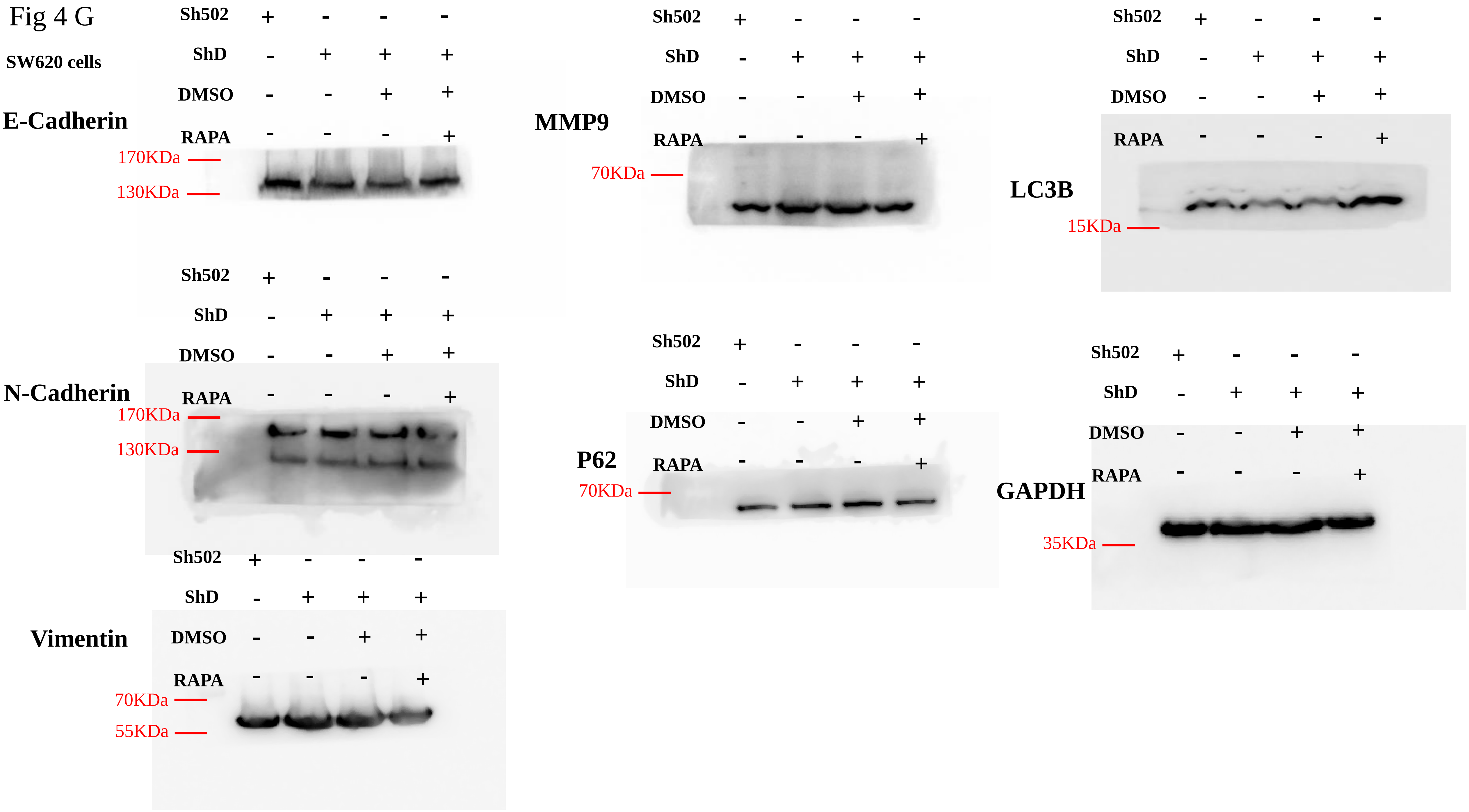
SW620 cells
Sh502 + - - -
ShD - + + +
DMSO - - + +
RAPA - - - +
E-Cadherin
170KDa
130KDa
N-Cadherin
170KDa
130KDa
Vimentin
70KDa
55KDa
MMP9
70KDa
P62
70KDa
LC3B
15KDa
GAPDH
35KDa
Fig 4 G

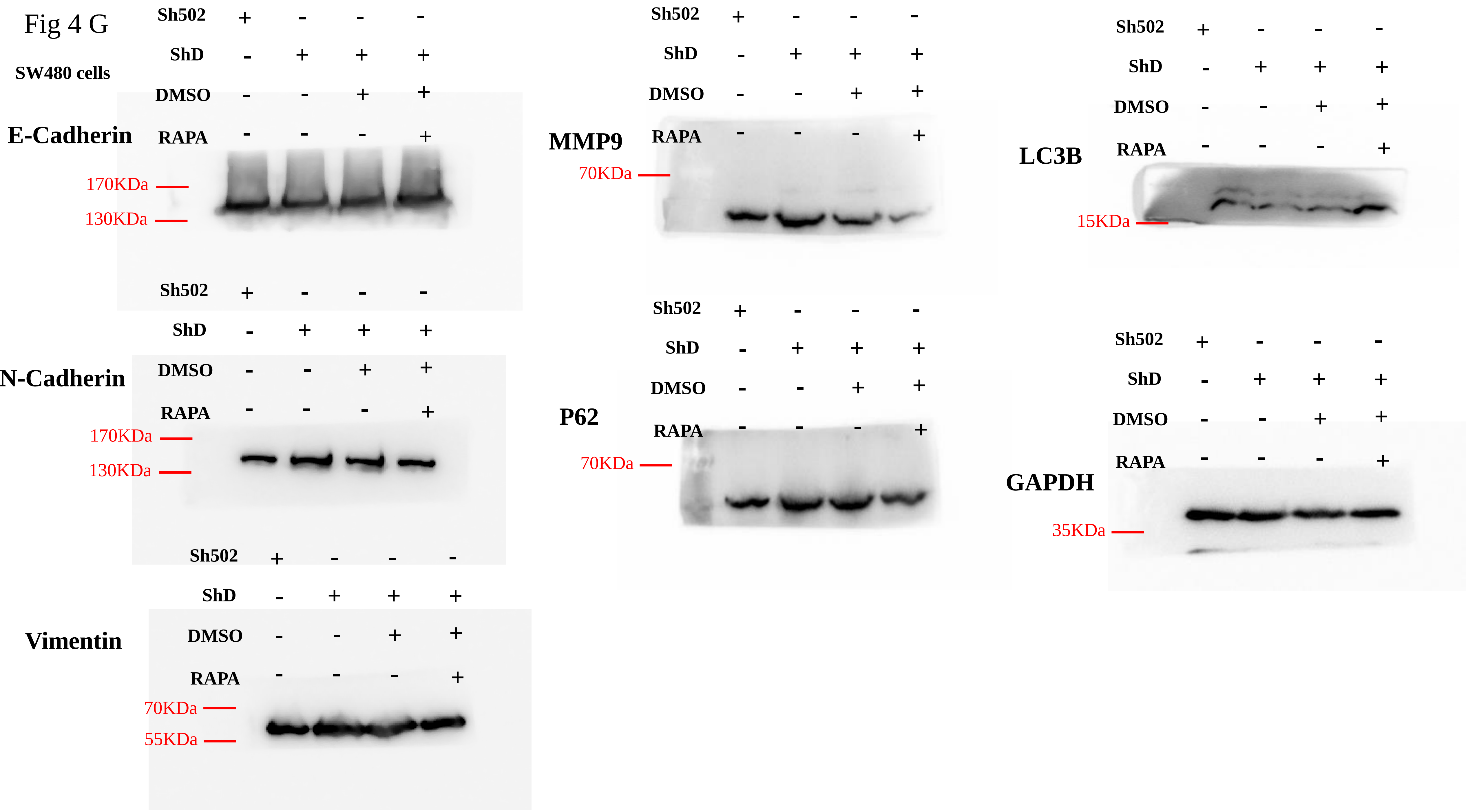

Fig 4 G
SW480 cells
Sh502 + - - -
ShD - + + +
DMSO - - + +
RAPA - - - +
E-Cadherin
170KDa
130KDa
N-Cadherin
170KDa
130KDa
Vimentin
70KDa
55KDa
MMP9
70KDa
P62
70KDa
LC3B
15KDa
GAPDH
35KDa

Fig 5 A

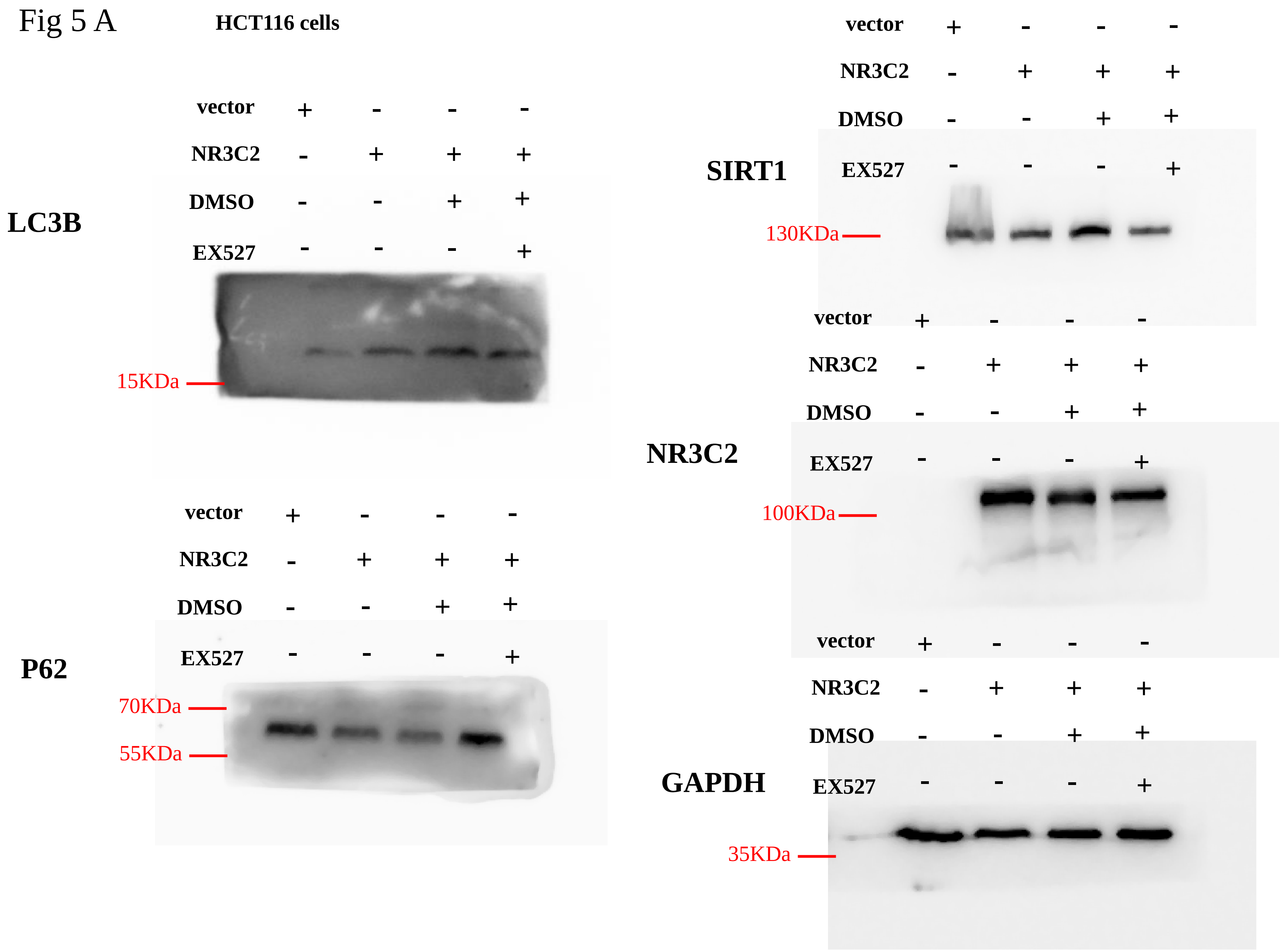
HCT116 cells
LC3B
vector + - - -
NR3C2 - + + +
DMSO - - + +
EX527 - - - +
15KDa
P62
vector + - - -
NR3C2 - + + +
DMSO - - + +
EX527 - - - +
70KDa
55KDa
SIRT1
vector + - - -
NR3C2 - + + +
DMSO - - + +
EX527 - - - +
130KDa
NR3C2
vector + - - -
NR3C2 - + + +
DMSO - - + +
EX527 - - - +
100KDa
GAPDH
vector + - - -
NR3C2 - + + +
DMSO - - + +
EX527 - - - +
35KDa

Fig 5 B

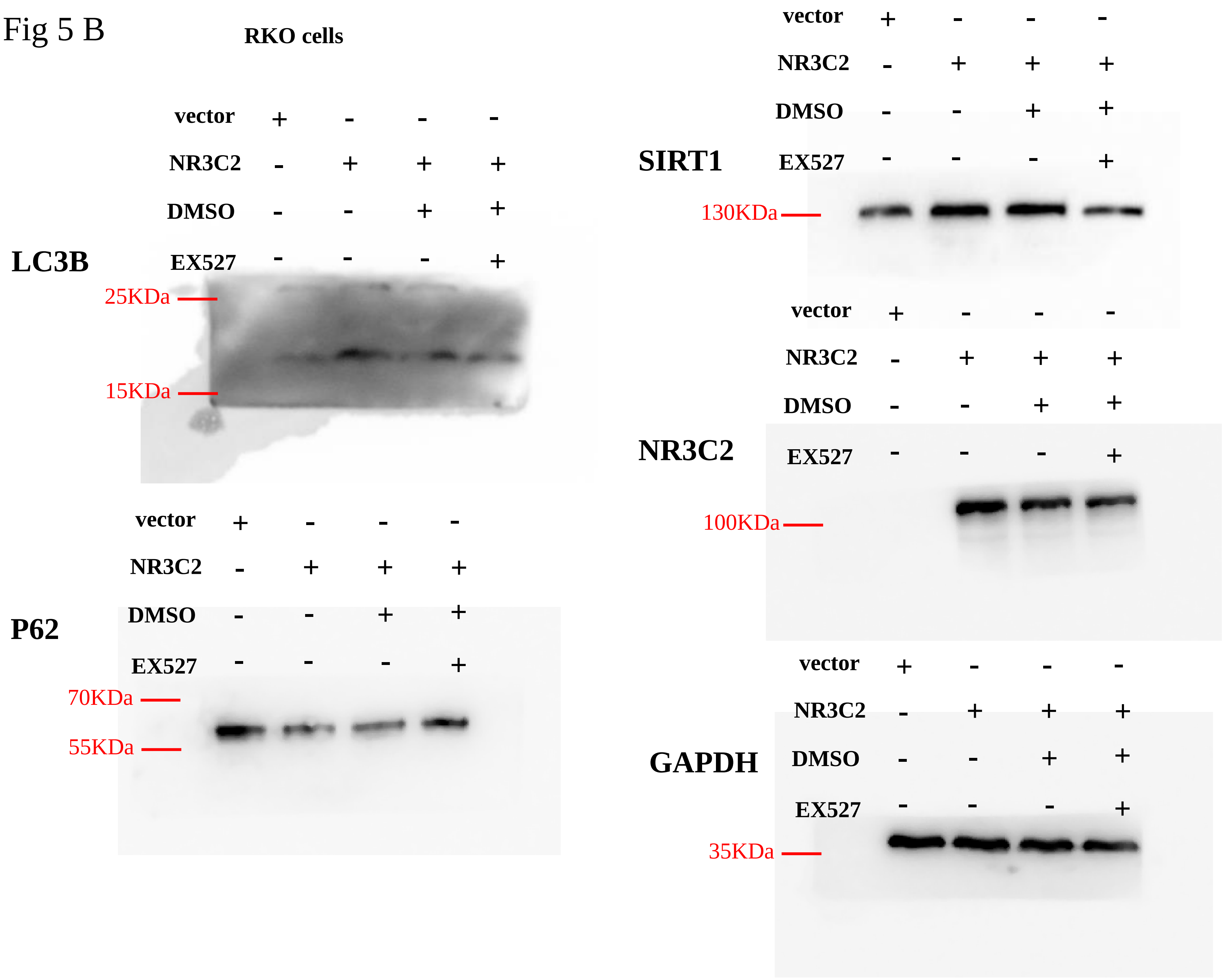

RKO cells
LC3B
vector + - - -
NR3C2 - + + +
DMSO - - + +
EX527 - - - +
25KDa
15KDa
P62
vector + - - -
NR3C2 - + + +
DMSO - - + +
EX527 - - - +
70KDa
55KDa
SIRT1
vector + - - -
NR3C2 - + + +
DMSO - - + +
EX527 - - - +
130KDa
NR3C2
vector + - - -
NR3C2 - + + +
DMSO - - + +
EX527 - - - +
100KDa
GAPDH
vector + - - -
NR3C2 - + + +
DMSO - - + +
EX527 - - - +
35KDa

Fig 5 C

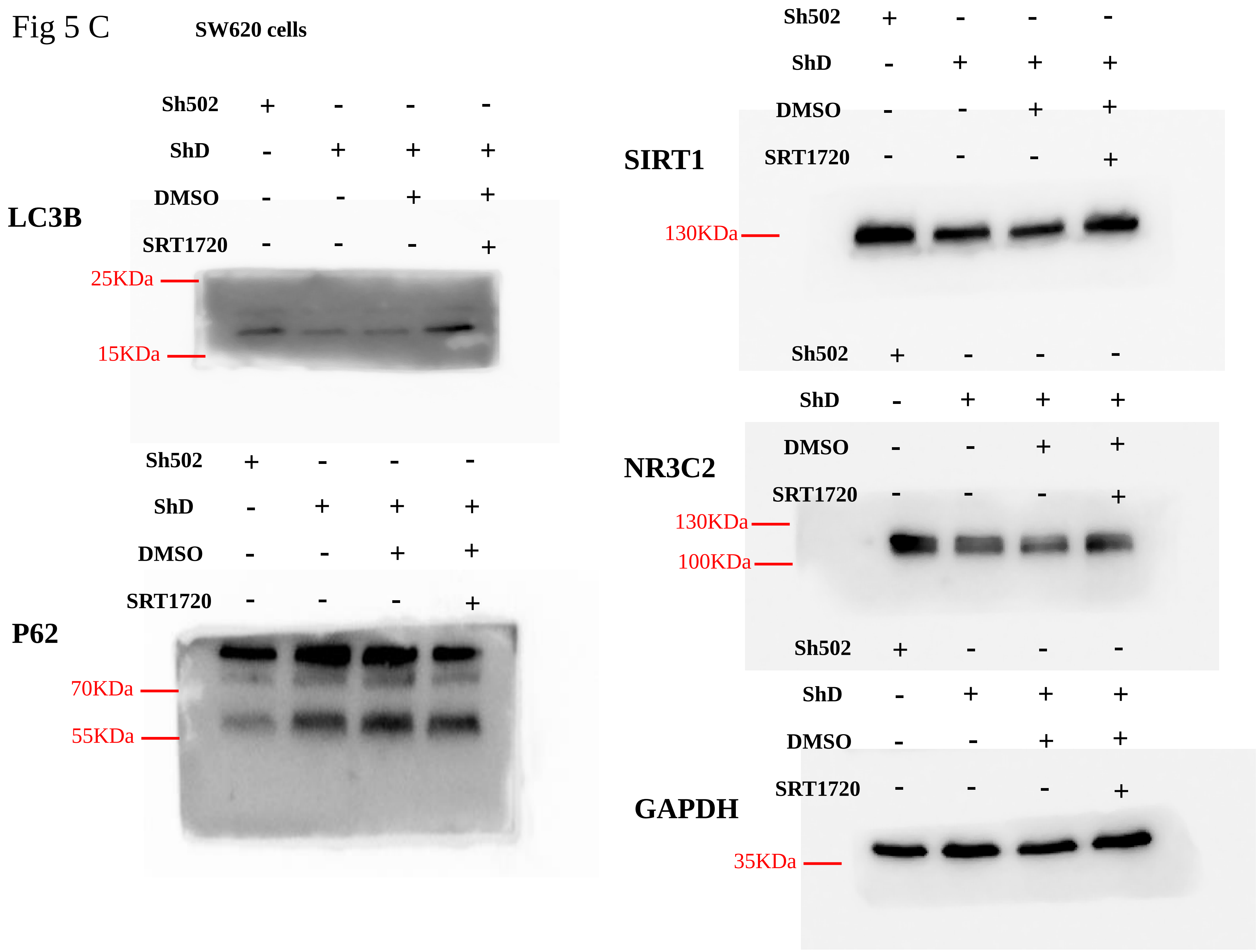
SW620 cells
LC3B
Sh502 + - - -
ShD - + + +
DMSO - - + +
SRT1720 - - - +
25KDa
15KDa
P62
Sh502 + - - -
ShD - + + +
DMSO - - + +
SRT1720 - - - +
70KDa
55KDa
SIRT1
Sh502 + - - -
ShD - + + +
DMSO - - + +
SRT1720 - - - +
130KDa
NR3C2
Sh502 + - - -
ShD - + + +
DMSO - - + +
SRT1720 - - - +
130KDa
100KDa
GAPDH
Sh502 + - - -
ShD - + + +
DMSO - - + +
SRT1720 - - - +
35KDa

Fig 5 D

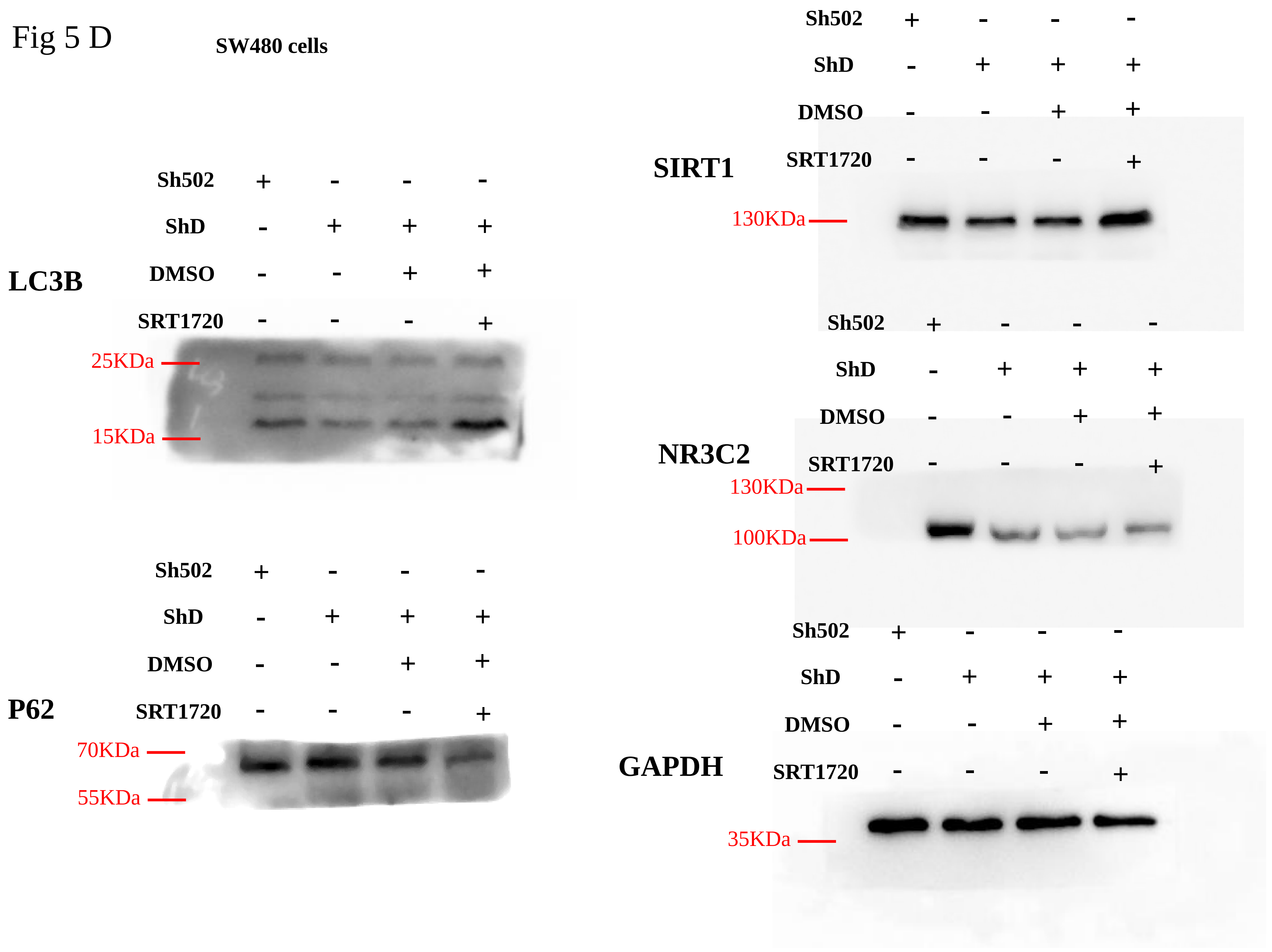
SW480 cells
LC3B
Sh502 + - - -
ShD - + + +
DMSO - - + +
SRT1720 - - - +
25KDa
15KDa
P62
Sh502 + - - -
ShD - + + +
DMSO - - + +
SRT1720 - - - +
70KDa
55KDa
SIRT1
Sh502 + - - -
ShD - + + +
DMSO - - + +
SRT1720 - - - +
130KDa
NR3C2
Sh502 + - - -
ShD - + + +
DMSO - - + +
SRT1720 - - - +
130KDa
100KDa
GAPDH
Sh502 + - - -
ShD - + + +
DMSO - - + +
SRT1720 - - - +
35KDa

Fig 5 E

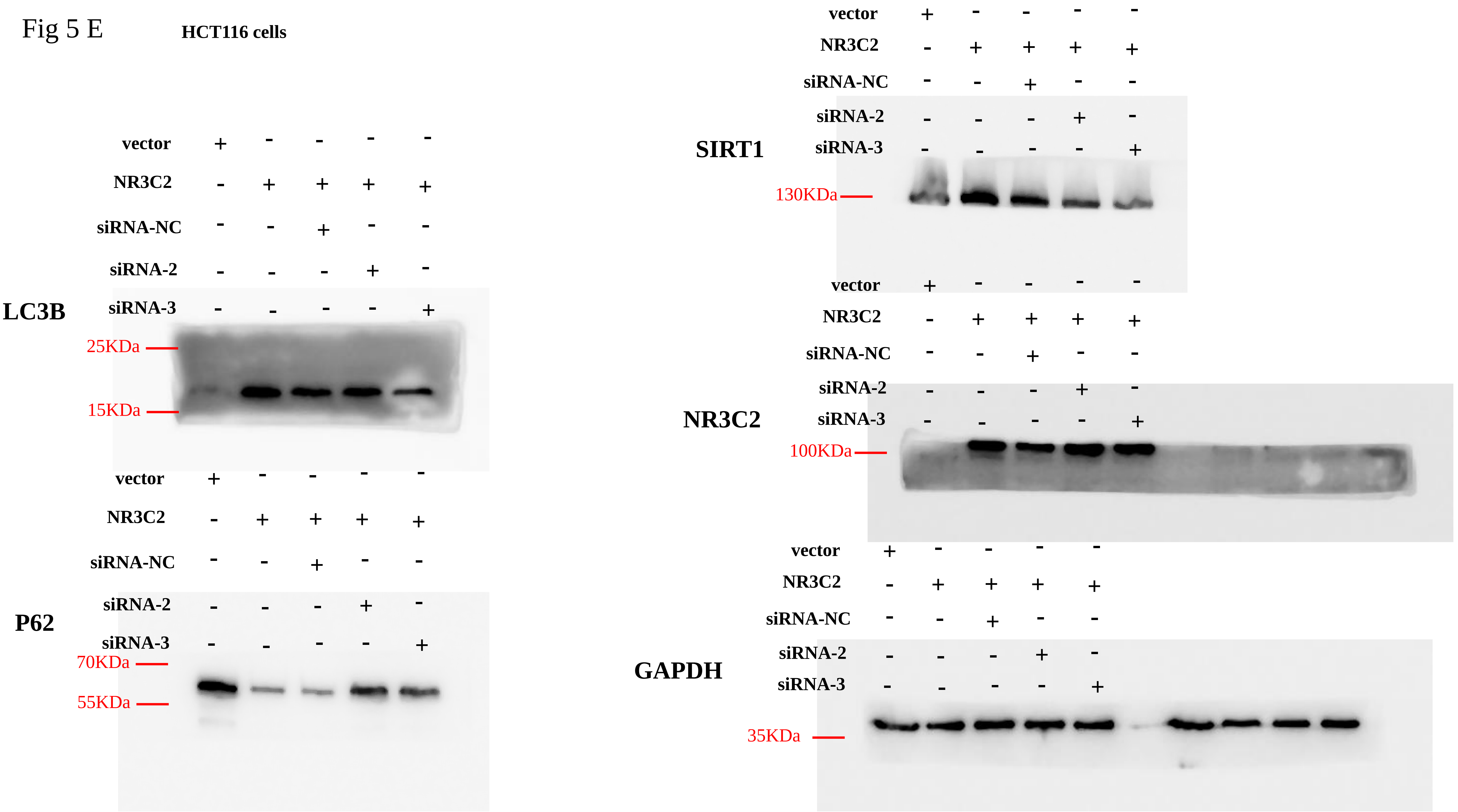
HCT116 cells
LC3B
vector + - - - -
NR3C2 - + + + +
siRNA-NC - - + - -
siRNA-2 - - - + -
siRNA-3 - - - - +
25KDa
15KDa
P62
vector + - - - -
NR3C2 - + + + +
siRNA-NC - - + - -
siRNA-2 - - - + -
siRNA-3 - - - - +
70KDa
55KDa
SIRT1
vector + - - - -
NR3C2 - + + + +
siRNA-NC - - + - -
siRNA-2 - - - + -
siRNA-3 - - - - +
130KDa
NR3C2
vector + - - - -
NR3C2 - + + + +
siRNA-NC - - + - -
siRNA-2 - - - + -
siRNA-3 - - - - +
100KDa
GAPDH
vector + - - - -
NR3C2 - + + + +
siRNA-NC - - + - -
siRNA-2 - - - + -
siRNA-3 - - - - +
35KDa

Fig 5 F

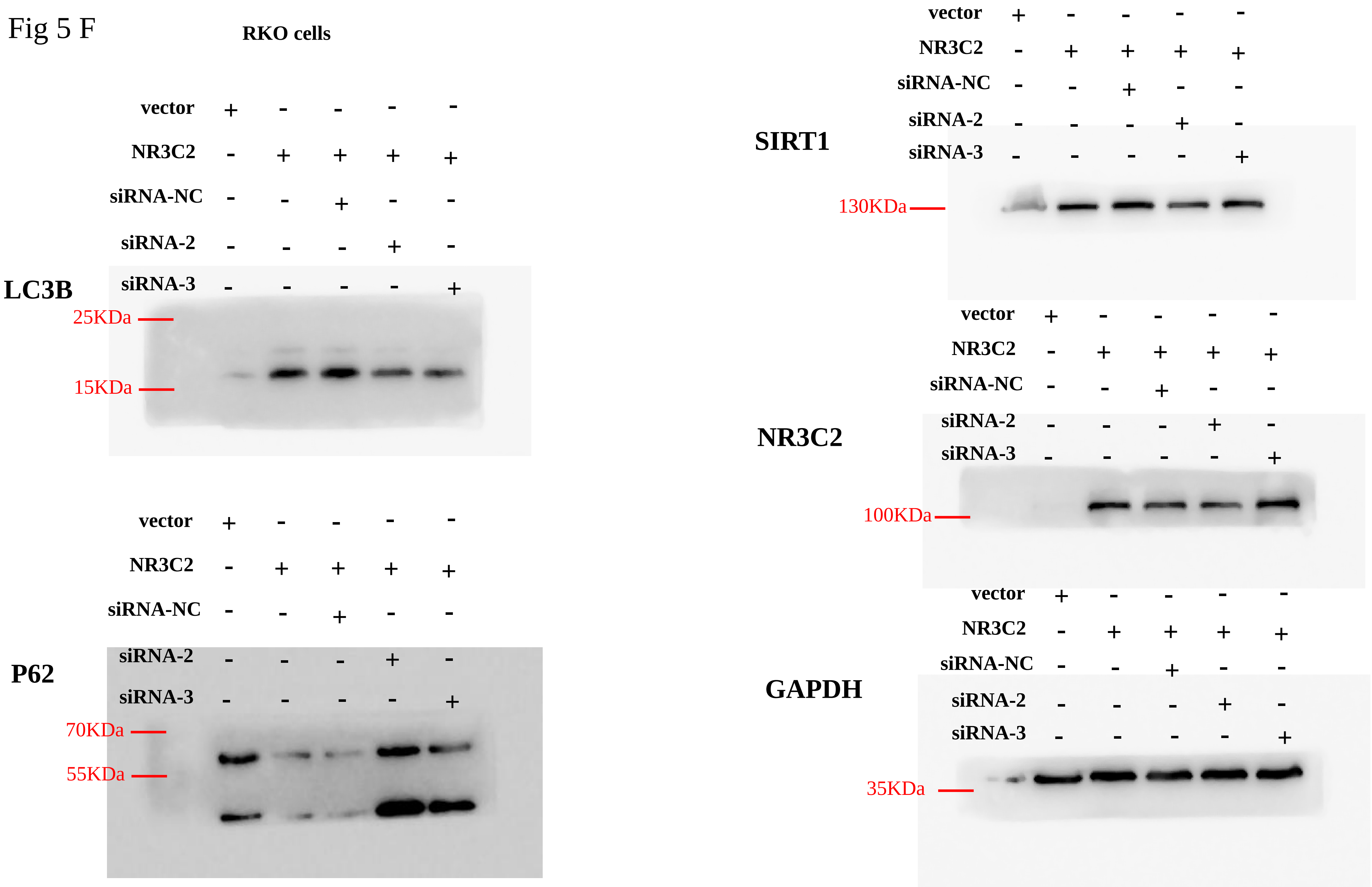
RKO cells
vector + - - - -
NR3C2 - + + + +
siRNA-NC - - + - -
siRNA-2 - - - + -
siRNA-3 - - - - +
LC3B
25KDa
15KDa
P62
70KDa
55KDa
SIRT1
130KDa
NR3C2
100KDa
GAPDH
35KDa

Fig 6 E

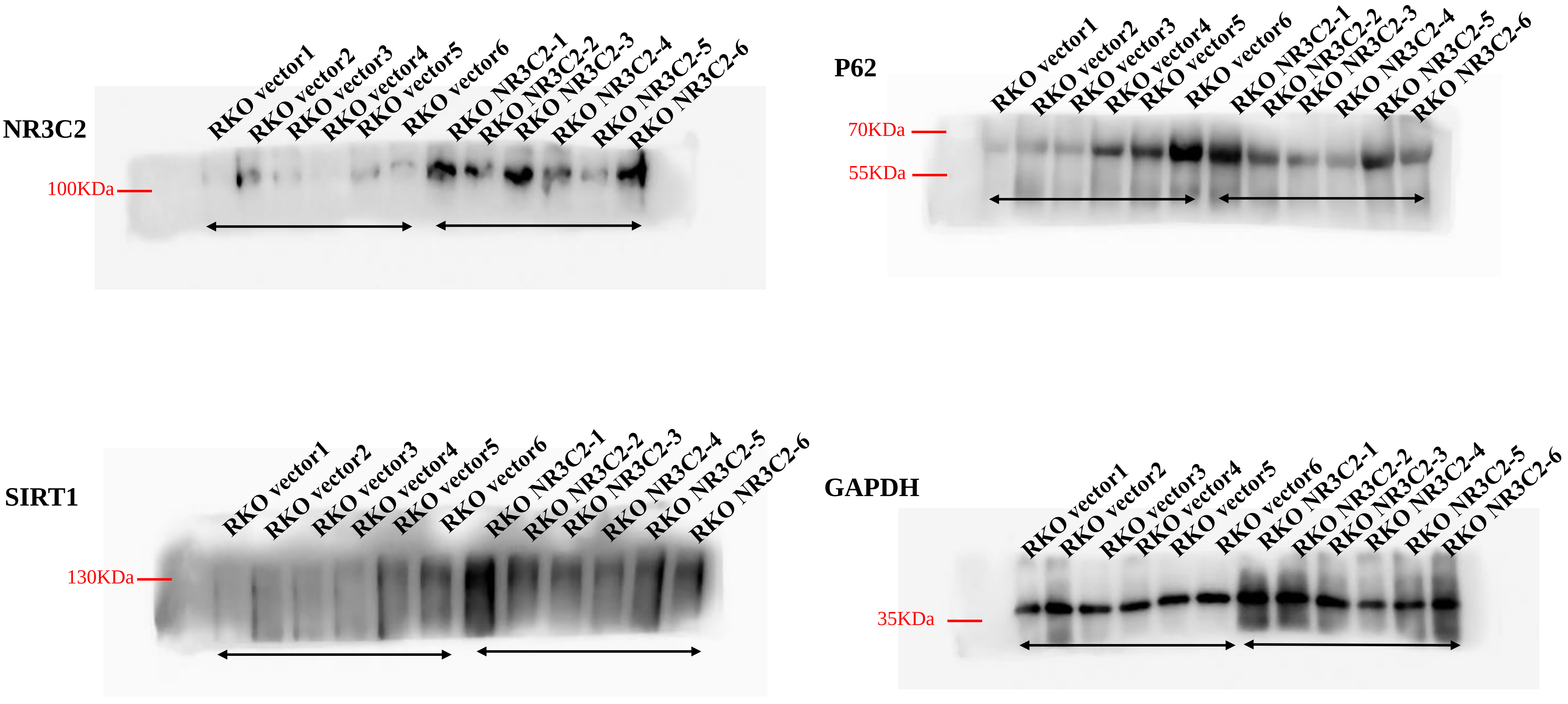
NR3C2
RKO vector1
RKO vector2
RKO vector3
RKO vector4
RKO vector5
RKO vector6
RKO NR3C2-1
RKO NR3C2-2
RKO NR3C2-3
RKO NR3C2-4
RKO NR3C2-5
RKO NR3C2-6
100KDa
P62
70KDa
55KDa
SIRT1
130KDa
GAPDH
35KDa

Fig 6 F

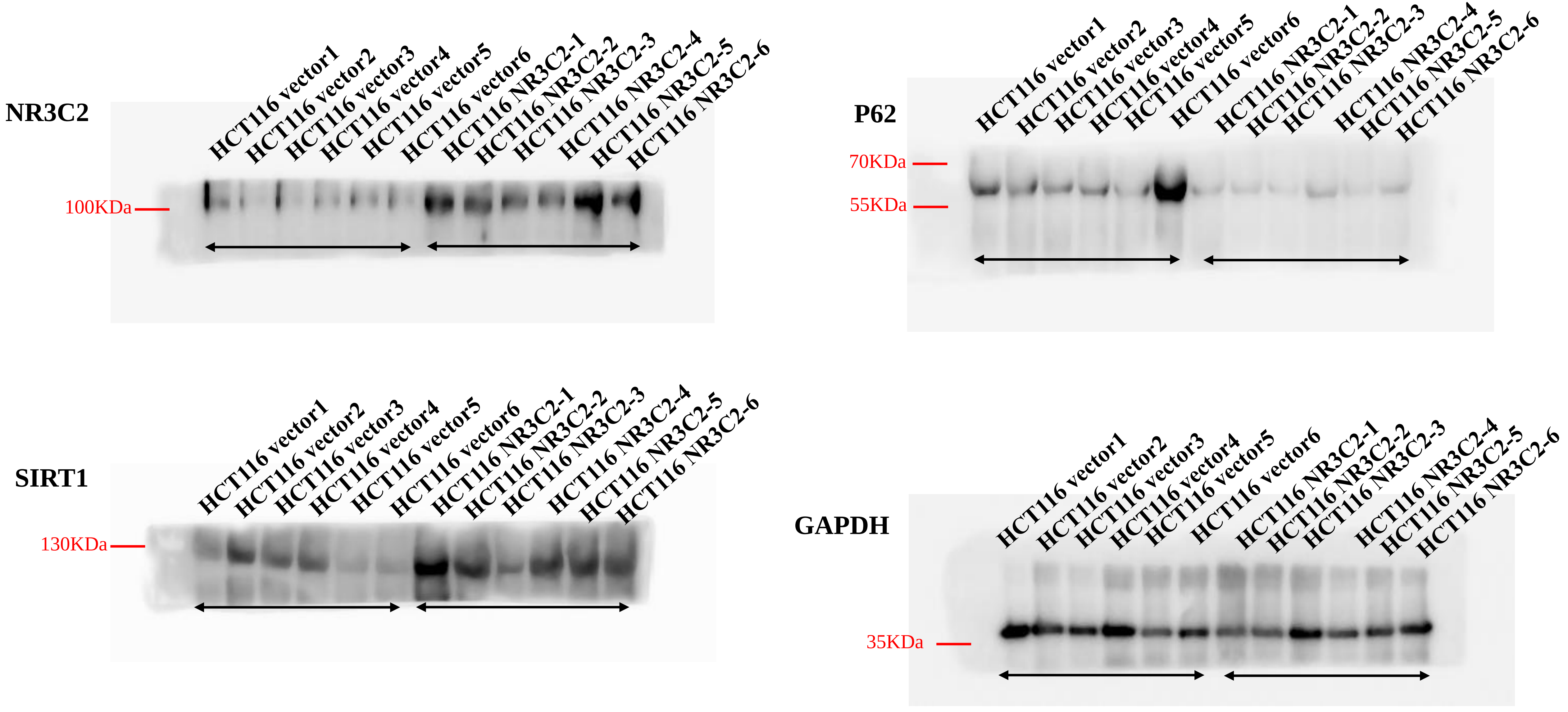
NR3C2
HCT116 vector1
HCT116 vector2
HCT116 vector3
HCT116 vector4
HCT116 vector5
HCT116 vector6
HCT116 NR3C2-1
HCT116 NR3C2-2
HCT116 NR3C2-3
HCT116 NR3C2-4
HCT116 NR3C2-5
HCT116 NR3C2-6
100KDa
P62
HCT116 vector1
HCT116 vector2
HCT116 vector3
HCT116 vector4
HCT116 vector5
HCT116 vector6
HCT116 NR3C2-1
HCT116 NR3C2-2
HCT116 NR3C2-3
HCT116 NR3C2-4
HCT116 NR3C2-5
HCT116 NR3C2-6
70KDa
55KDa
SIRT1
HCT116 vector1
HCT116 vector2
HCT116 vector3
HCT116 vector4
HCT116 vector5
HCT116 vector6
HCT116 NR3C2-1
HCT116 NR3C2-2
HCT116 NR3C2-3
HCT116 NR3C2-4
HCT116 NR3C2-5
HCT116 NR3C2-6
130KDa
GAPDH
HCT116 vector1
HCT116 vector2
HCT116 vector3
HCT116 vector4
HCT116 vector5
HCT116 vector6
HCT116 NR3C2-1
HCT116 NR3C2-2
HCT116 NR3C2-3
HCT116 NR3C2-4
HCT116 NR3C2-5
HCT116 NR3C2-6
35KDa

Fig S1 B

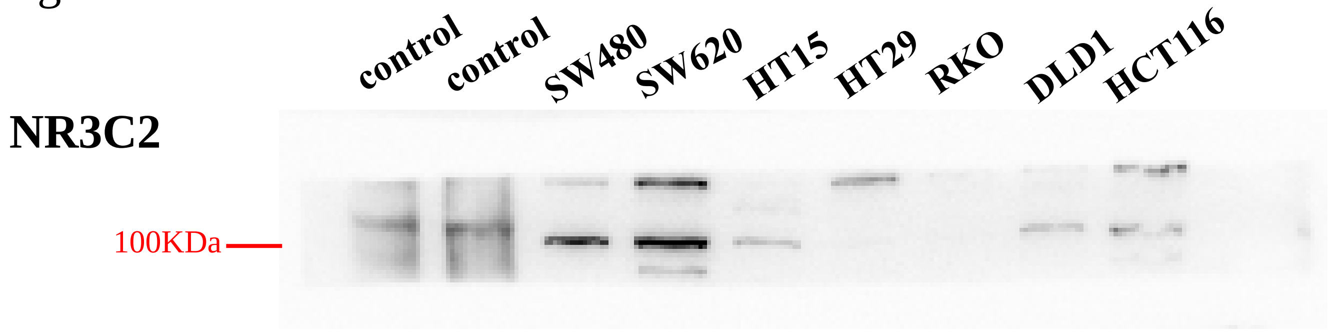
control
control
SW480
SW620
HT15
HT29
RKO
DLD1
HCT116
NR3C2
100KDa

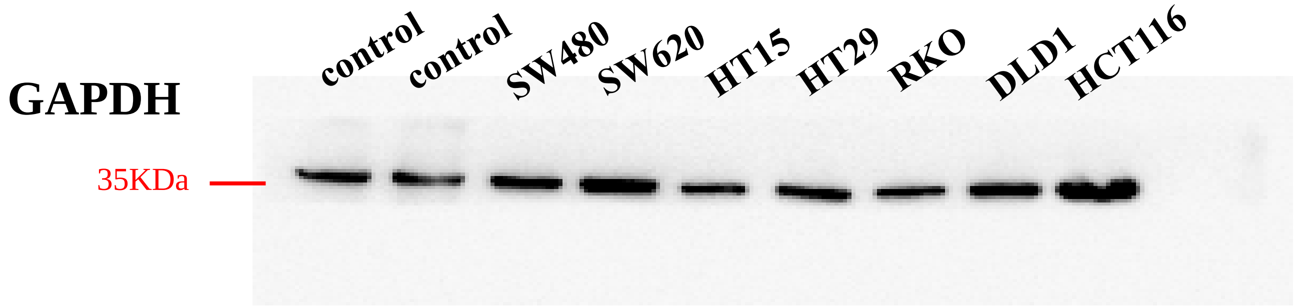
control
control
SW480
SW620
HT15
HT29
RKO
DLD1
HCT116
GAPDH
35KDa

Fig S1 D

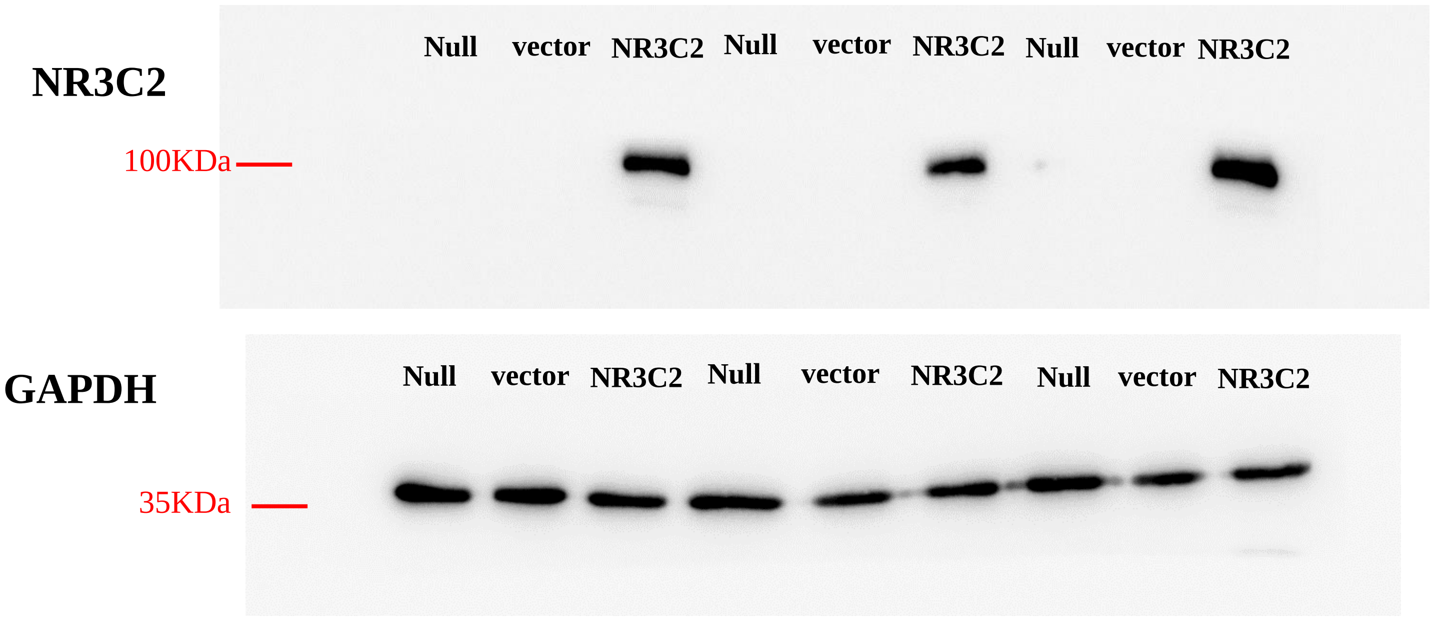

Fig S1 E

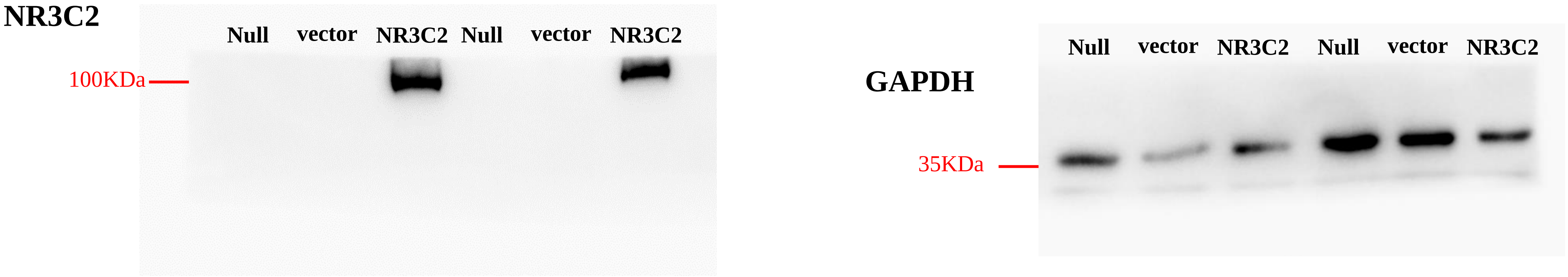

Fig S1 H

SW480 cells SW620 cells

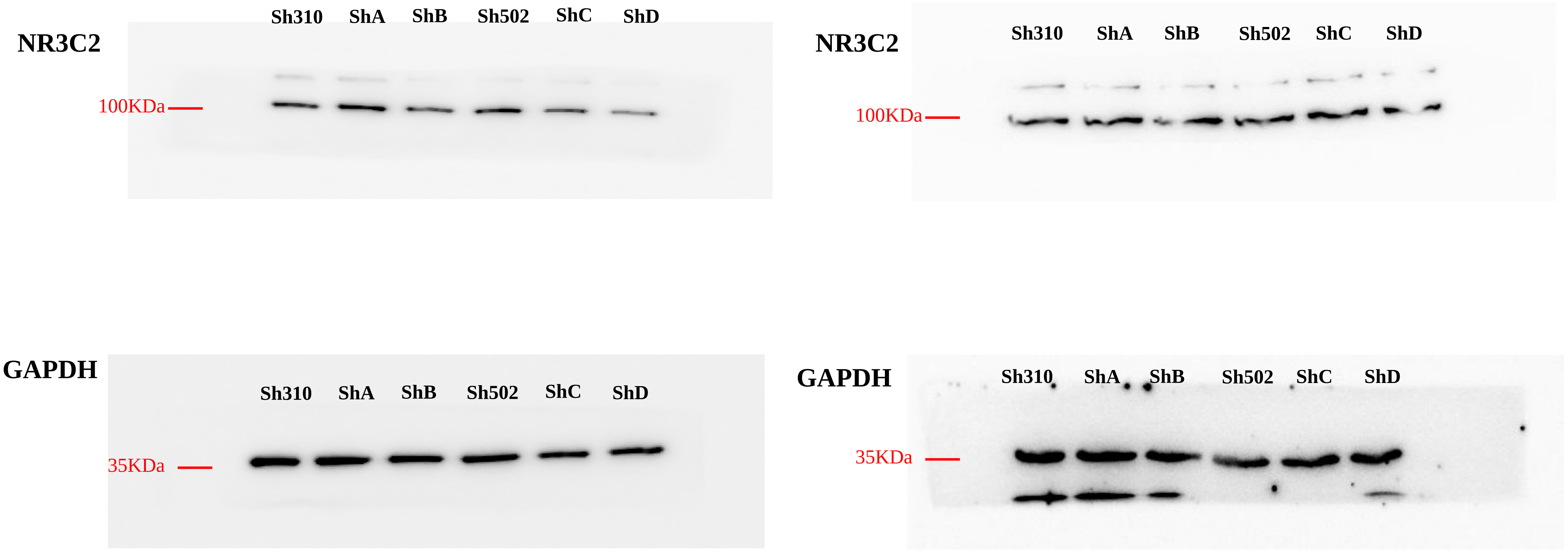

Fig S2 B

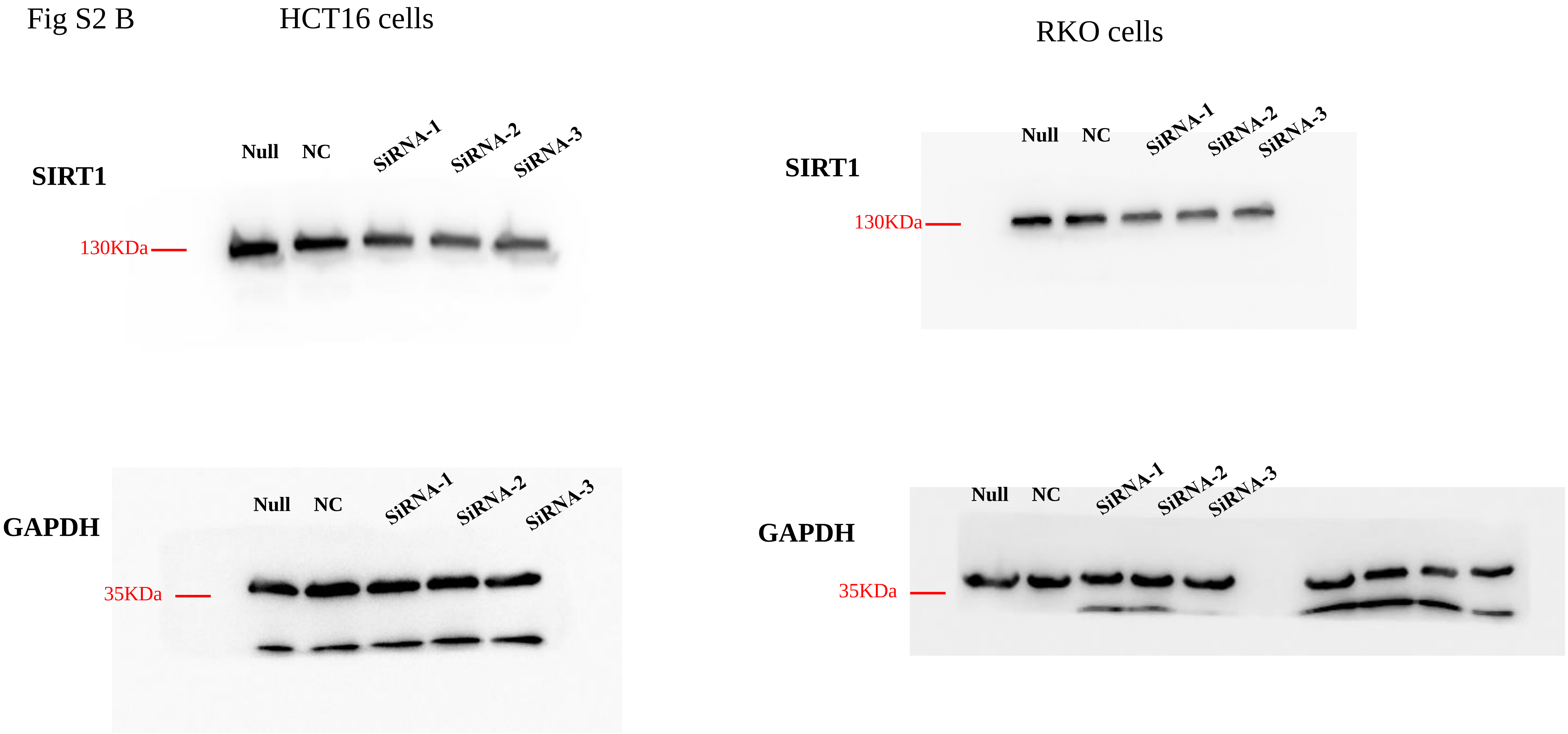
HCT16 cells
RKO cells
SIRT1
Null
NC
SiRNA-1
SiRNA-2
SiRNA-3
130KDa
SIRT1
Null
NC
SiRNA-1
SiRNA-2
SiRNA-3
130KDa
GAPDH
Null
NC
SiRNA-1
SiRNA-2
SiRNA-3
35KDa
GAPDH
Null
NC
SiRNA-1
SiRNA-2
SiRNA-3
35KDa

Fig S3 A

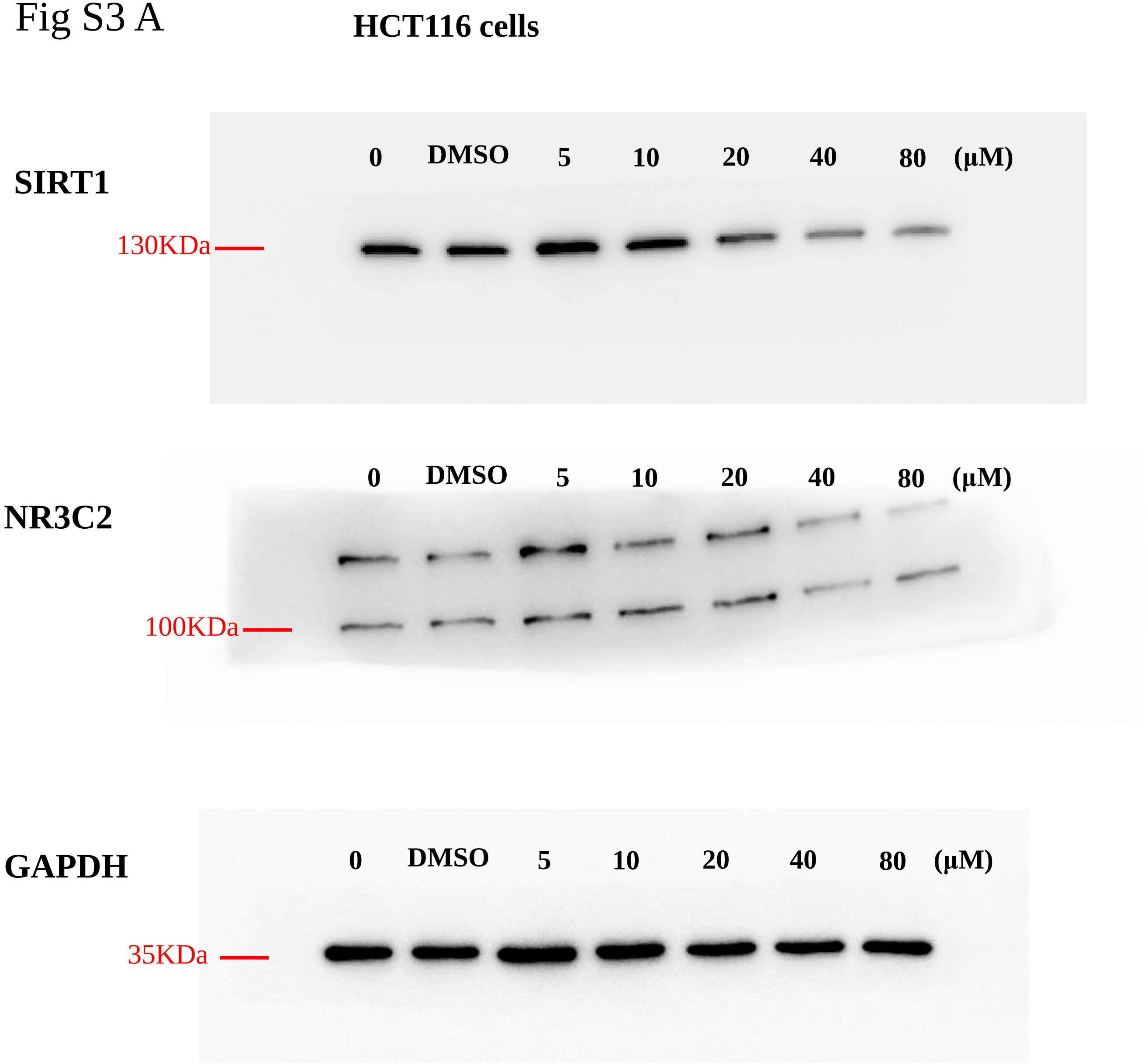
HCT116 cells
SIRT1
0
DMSO
5
10
20
40
80
(μM)
130KDa
NR3C2
0
DMSO
5
10
20
40
80
(μM)
100KDa
GAPDH
0
DMSO
5
10
20
40
80
(μM)
35KDa

Fig S3 B

RKO cells

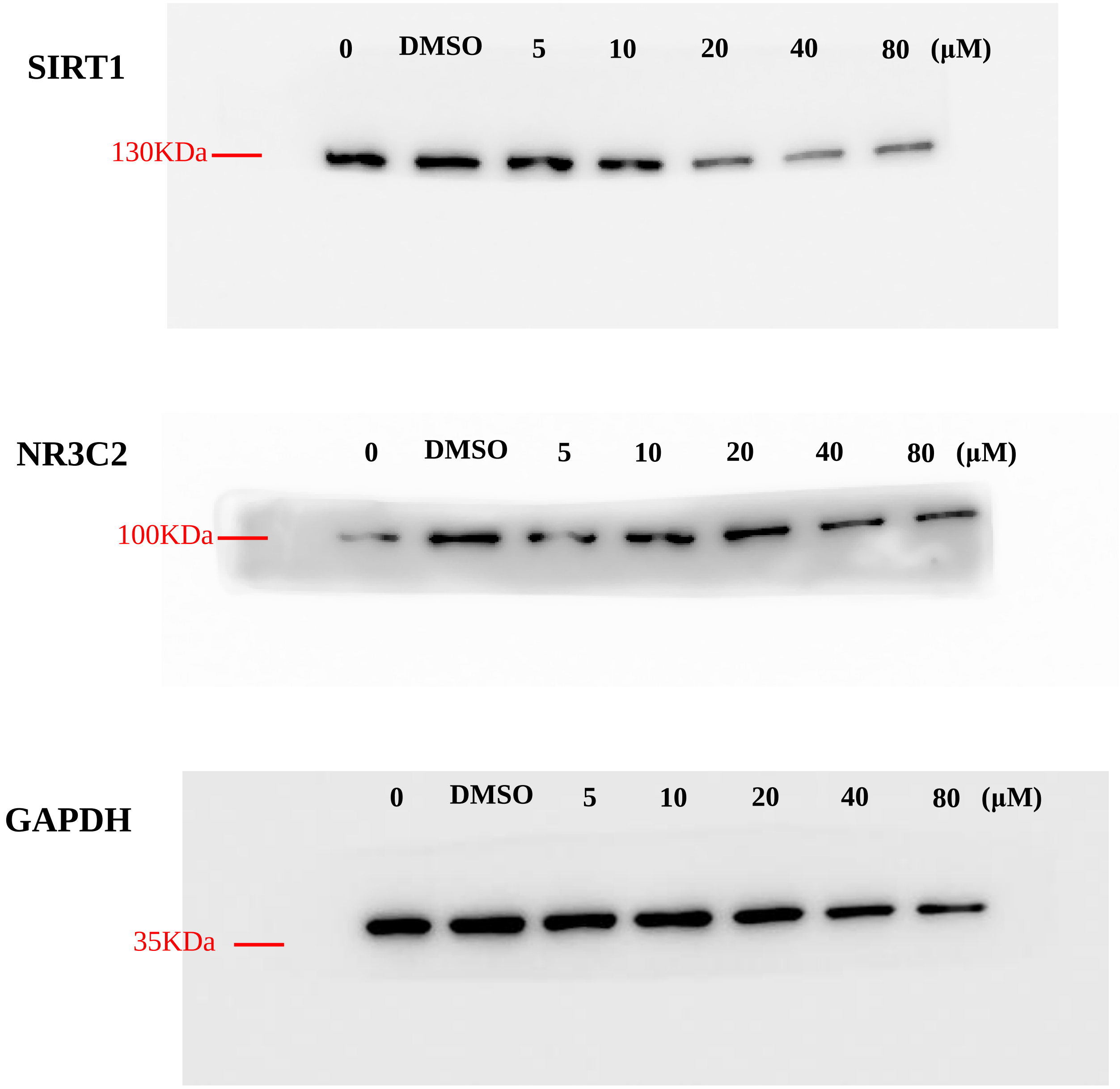

Fig S3 C

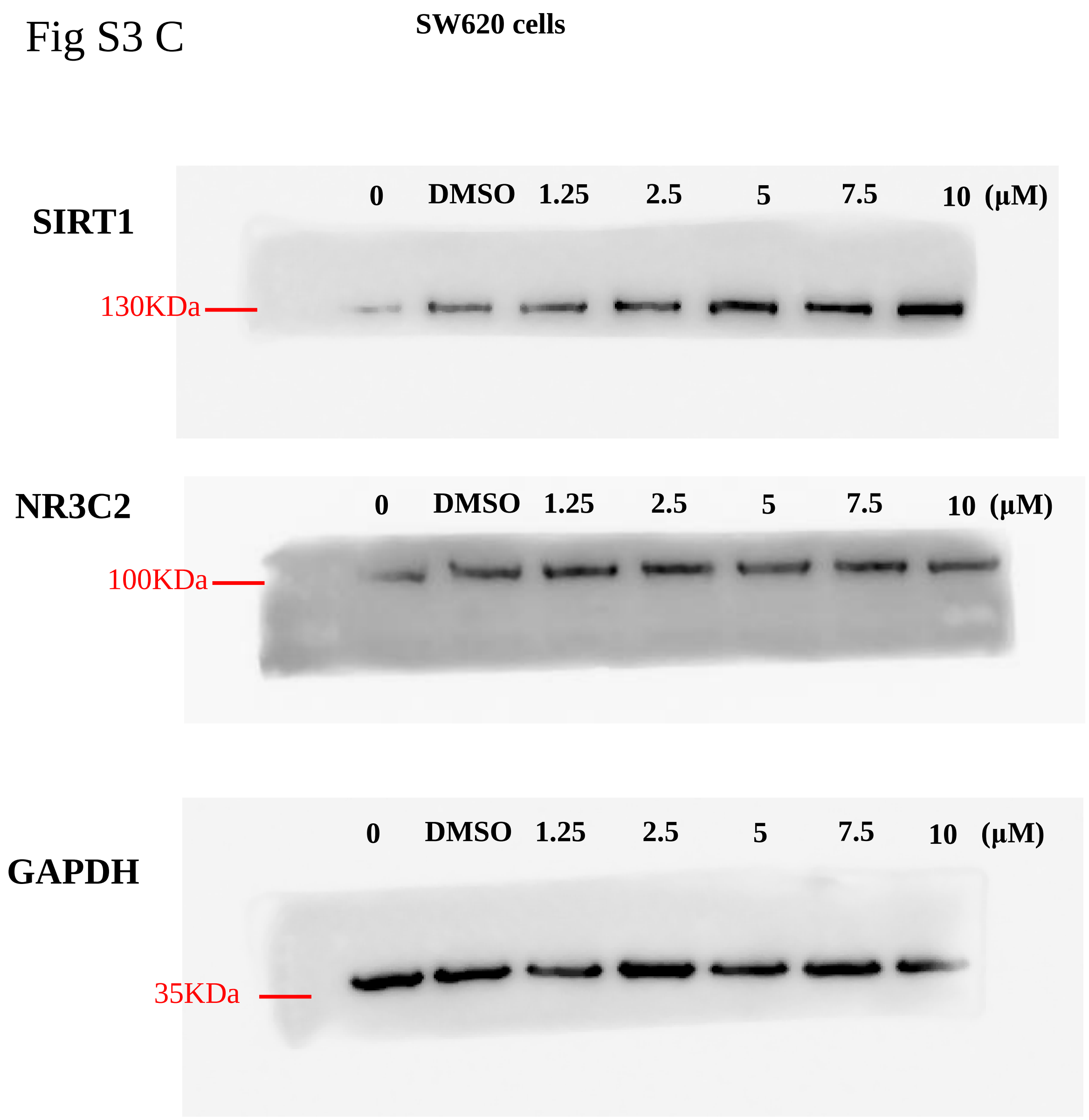

SW620 cells
SIRT1
0 DMSO 1.25 2.5 5 7.5 10 (μM)
130KDa
NR3C2
0 DMSO 1.25 2.5 5 7.5 10 (μM)
100KDa
GAPDH
0 DMSO 1.25 2.5 5 7.5 10 (μM)
35KDa

Fig S3 D

SW480 cells

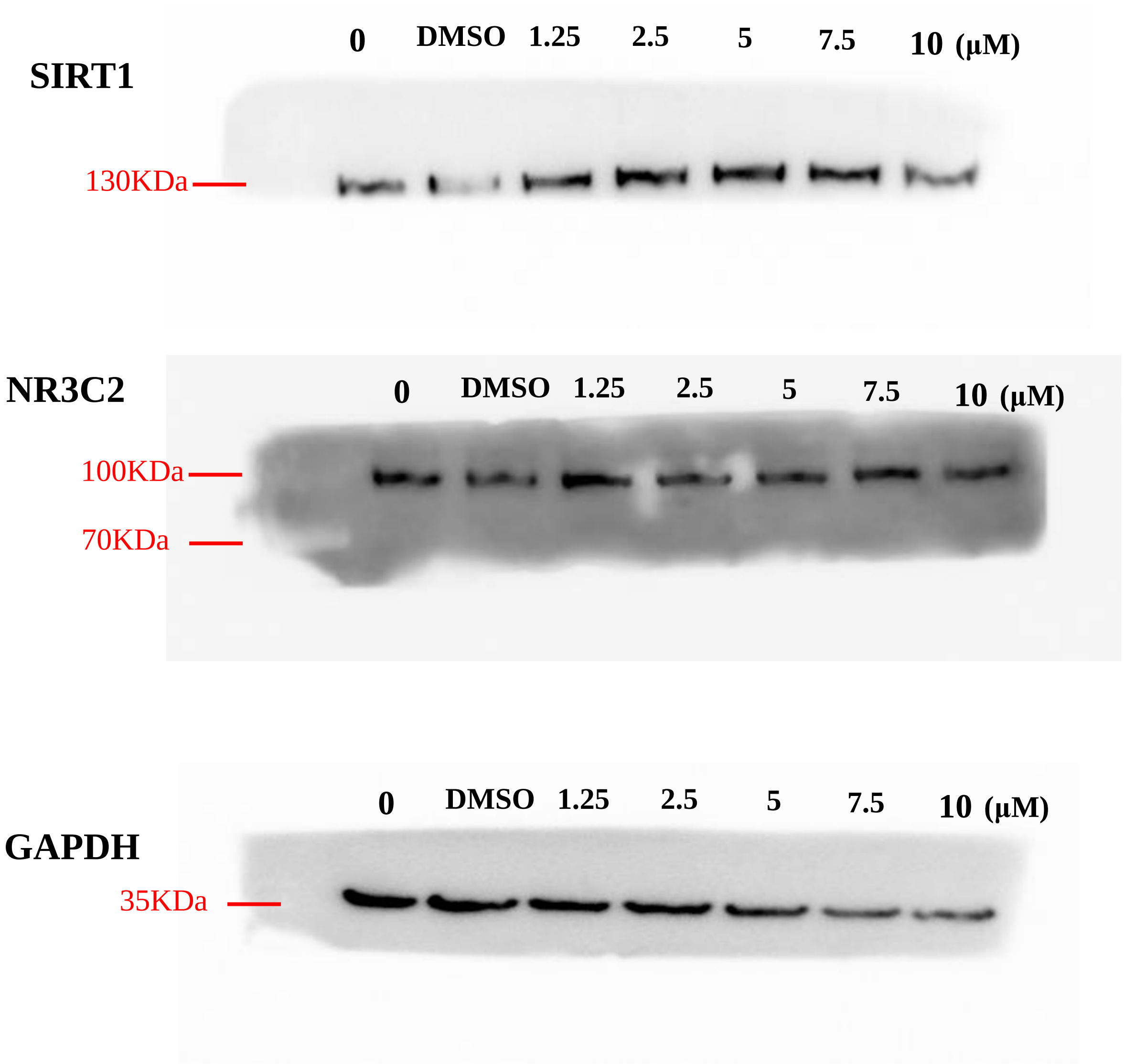

Fig S7 D

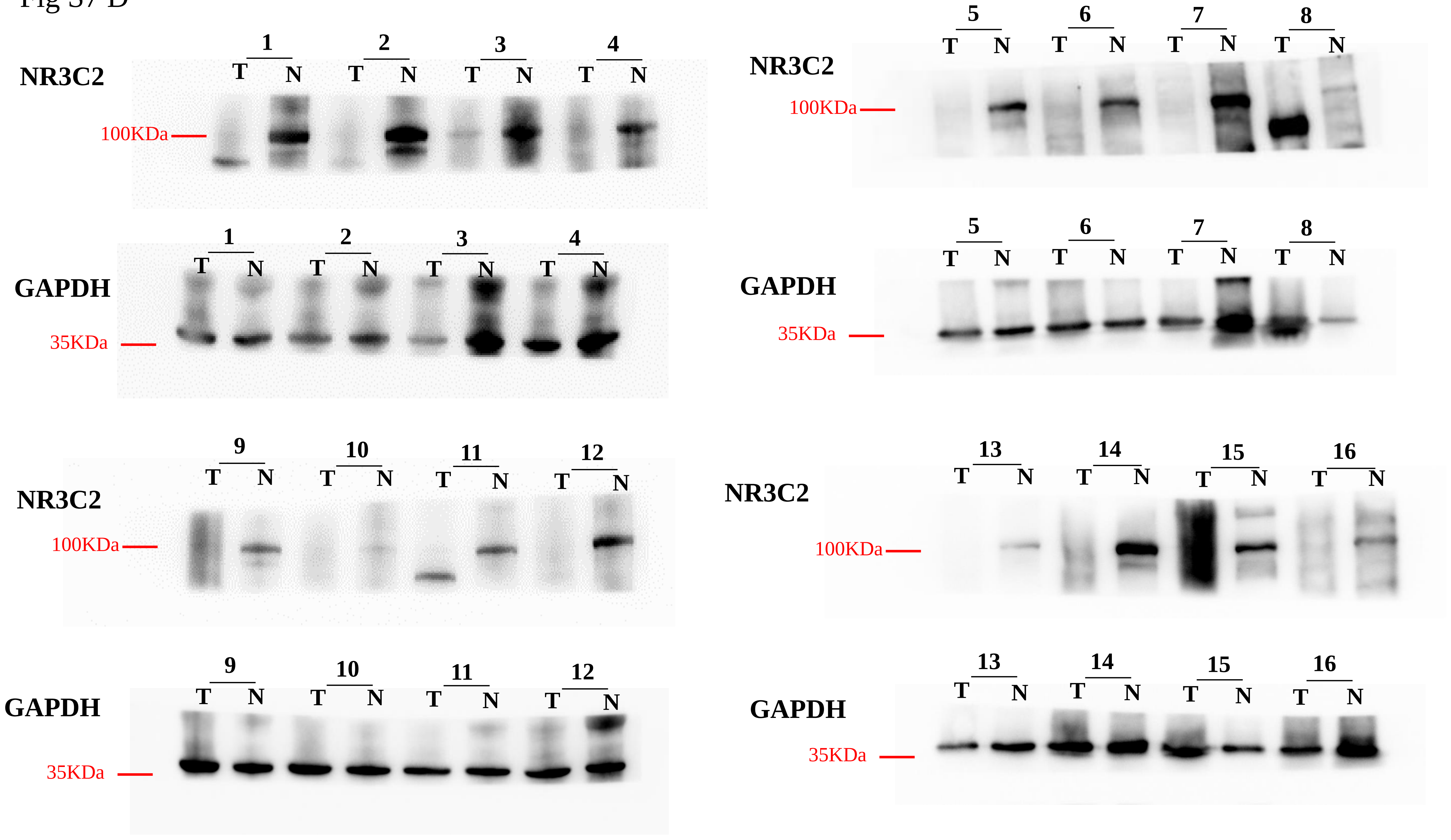

Fig S7 D

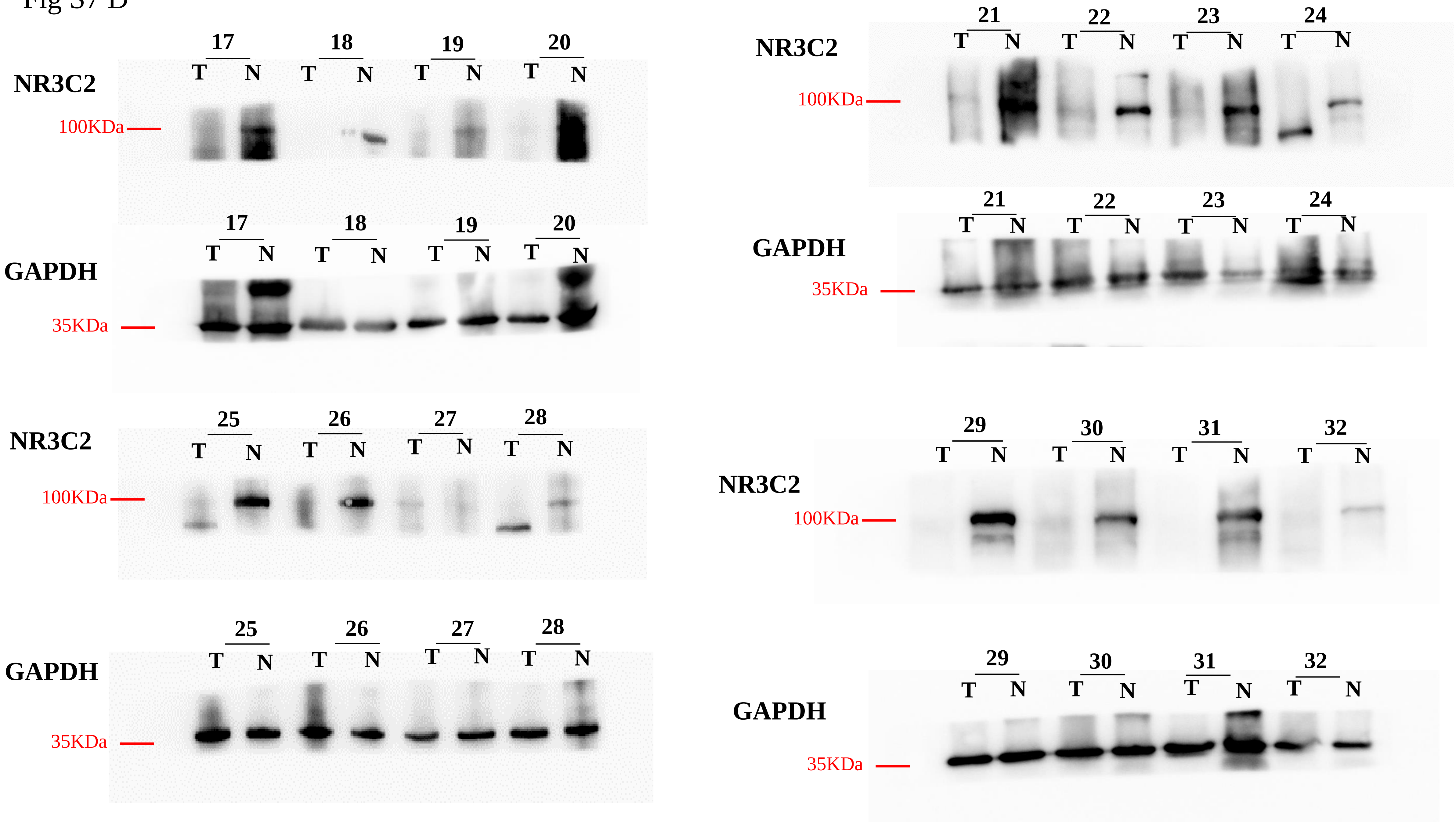
NR3C2
17 18 19 20
T N T N T N T N
100KDa
GAPDH
17 18 19 20
T N T N T N T N
35KDa
NR3C2
21 22 23 24
T N T N T N T N
100KDa
GAPDH
21 22 23 24
T N T N T N T N
35KDa
NR3C2
25 26 27 28
T N T N T N T N
100KDa
GAPDH
25 26 27 28
T N T N T N T N
35KDa
NR3C2
29 30 31 32
T N T N T N T N
100KDa
GAPDH
29 30 31 32
T N T N T N T N
35KDa

Fig S8 E

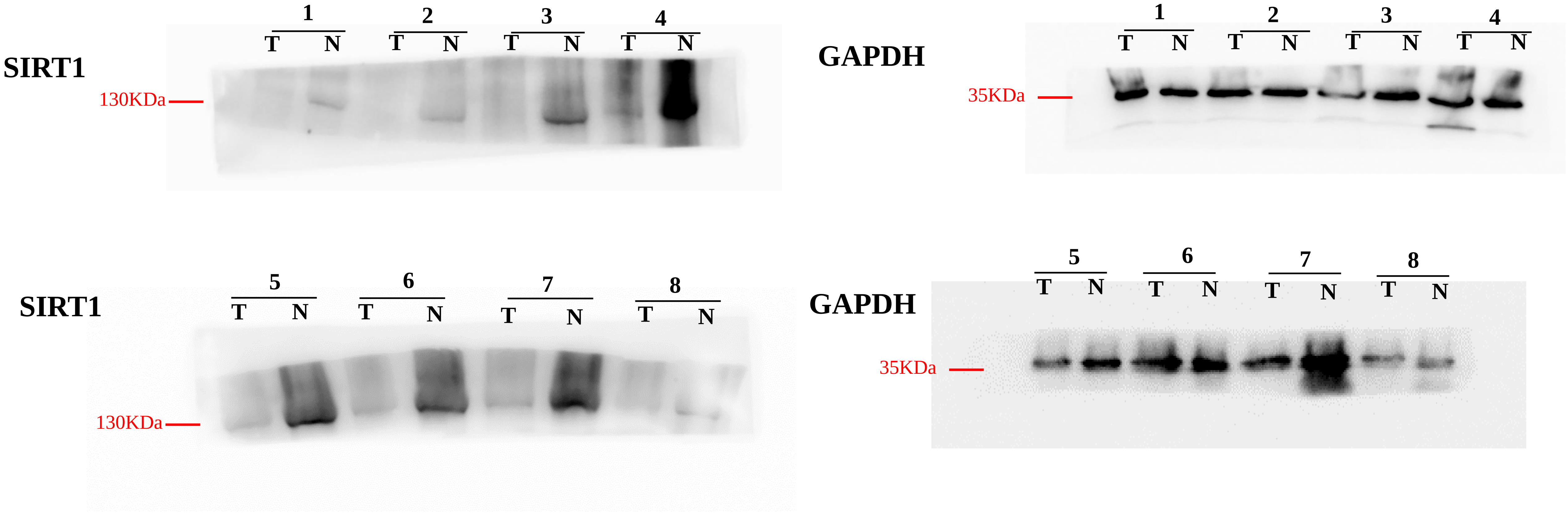
SIRT1
1 2 3 4
T N T N T N T N
130KDa
GAPDH
1 2 3 4
T N T N T N T N
35KDa
SIRT1
5 6 7 8
T N T N T N T N
130KDa
GAPDH
5 6 7 8
T N T N T N T N
35KDa

Fig S8 E

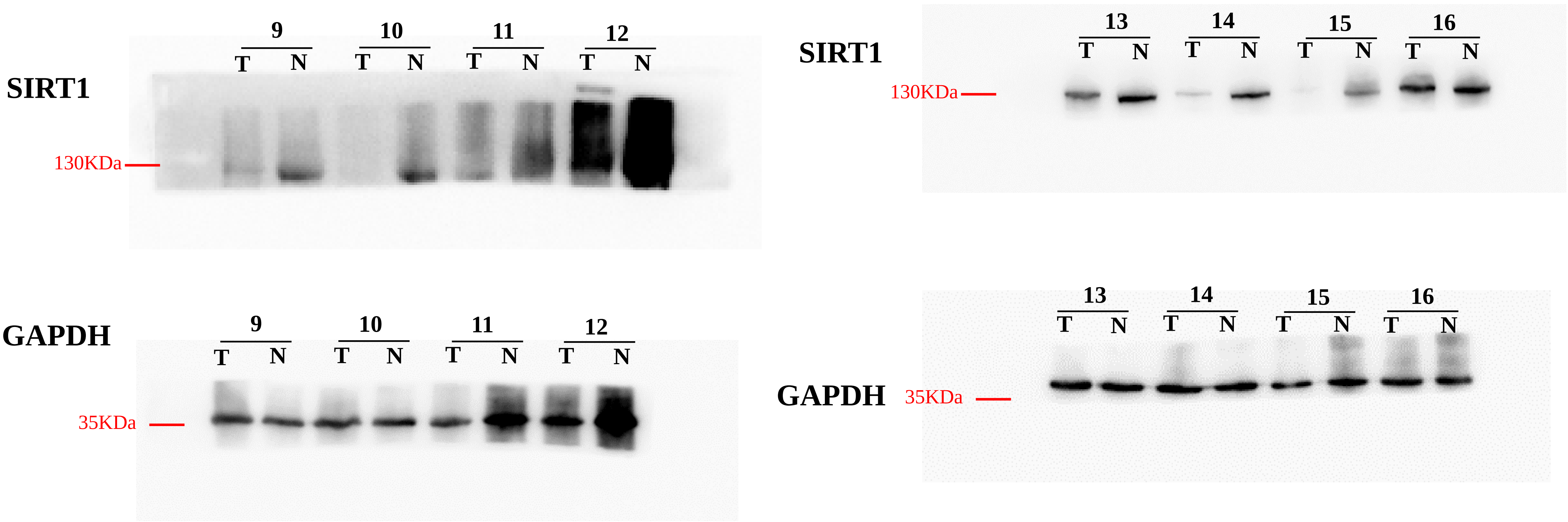
SIRT1
9
10
11
12
T N T N T N T N
130KDa
SIRT1
13
14
15
16
T N T N T N T N
130KDa
GAPDH
9
10
11
12
T N T N T N T N
35KDa
GAPDH
13
14
15
16
T N T N T N T N
35KDa
