# Supplementary material for: The NR3C2-SIRT1 signaling axis promotes autophagy and inhibits epithelial mesenchymal transition in colorectal cancer
Source: Cell Death Dis. 2025 Apr 14;16(1):295. doi: 10.1038/s41419-025-07575-3 (PMC11997134; doi:10.1038/s41419-025-07575-3)
Supplement: Supplementary file 1 — The NR3C2-SIRT1 signaling axis promotes autophagy and inhibits epithelial mesenchymal transition in colorectal cancer [file 41419_2025_7575_MOESM1_ESM.docx]

The NR3C2-SIRT1 signaling axis promotes autophagy and inhibits epithelial mesenchymal transition in colorectal cancer

Feng Li^1^, Xing Wan^1^, Zhigui Li^2*^, Liming Zhou^1*^

1) Department of Pharmacology, West China School of Basic Science and Forensic Medicine, Sichuan University, Chengdu, China.

2) Department of General Surgery, West China Hospital, Sichuan University.

*Corresponding author. Email: lizhigui07@sina.com; zhou108@163.com

Fig S1 Selection of CRC cells and cells transfection. (A): qRT-PCR detection of NR3C2 expression in 7 cell lines, ***P* < 0.01, ****P* < 0.001; (B): Western blot detection of NR3C2 expression in 7 cell lines, ***P* < 0.01, ****P* < 0.001; (C): Fluorescence images of HCT116 and RKO cells after NR3C2 overexpression transfection; (D): qRT-PCR and Western blot detected the transfection efficiency of NR3C2 overexpression in HCT116 cells, upper: qRT-PCR, lower: western blot , ****P* < 0.001; (E): qRT-PCR and Western blot detected the transfection efficiency of NR3C2 overexpression in RKO cells, upper: qRT-PCR, lower: western blot , ****P* < 0.001; (F): Fluorescence images of SW620 and SW480 cells after NR3C2 knockdown transfection. (G): PCR detection of the transfection efficiency of NR3C2 knockdown in cells, left: SW620, right: SW480, **P* < 0.05; (H): Western blot detection of NR3C2 knockdown transfection efficiency in cells, upper: SW620, lower: SW480, **P* < 0.05.

Fig S2 siRNA transfection of SIRT1 knockdown efficiency detection. (A): qRT-PCR detection of the transfection efficiency of SIRT1 in CRC cells, **P* < 0.05; (B): Western blot detection of SIRT1 transfection efficiency in cells, upper: HCT116, lower: RKO, **P* < 0.05.

Fig S3 Dose exploration of SIRT1 inhibitors and activators, and the impact of SIRT1 on CRC cells migration. (A): Western blot detection of changes in SIRT1 and NR3C2 expression in HCT116 after adding EX527, **P* < 0.05; (B): Western blot detection of changes in SIRT1 and NR3C2 expression in RKO after adding EX527, **P* < 0.05; (C): Western blot detection of changes in SIRT1 and NR3C2 expression in SW620 after adding SRT1720, **P* < 0.05; (D): Western blot detection of changes in SIRT1 and NR3C2 expression in SW480 after adding SRT1720, **P* < 0.05; (E): Transwell migration experiment validating that EX527 increases CRC cells migration. Upper: HCT116, lower: RKO, **P* < 0.05(HCT116), #*P* < 0.05(RKO); (F): Transwell migration experiment validating that SRT1720 decreases CRC cell migration. Upper: SW620, lower: SW480, **P* < 0.05(SW620), #*P* < 0.05(SW480).

Fig S4 The GSEA profile for NR3C2 overexpression of RNA-seq.

Fig S5 Photos of mice using RKO cells to construct a mouse lung metastasis model.

Fig S6 Photos of mice using HCT116 cells to construct a mouse lung metastasis model.

Fig S7 NR3C2 was downregulation in the CRC tissues. (A): The correlation between the expression of NR3C2 and nodal metastasis, upper: COAD, lower: READ; (B): The relationship between NR3C2 expression and stage classification, upper: COAD, lower: READ; (C): Immunohistochemical detection of NR3C2 expression in patient tissue samples, left: tumor tissue, right: adjacent tissue(50X and 400X); (D): NR3C2 expression detected by Western blot in 32 patient tumor and adjacent non-cancerous tissues samples, bottom: grayscale statistics, **P* < 0.05; (E): qRT-PCR detection of NR3C2 expression and its correlation with age, gender, T, N, M staging, and clinical stage, **P* < 0.05, ***P* < 0.01.

Fig S8 SIRT1 was downregulated in CRC tissues. (A): TCGA database detection of SIRT1 expression across various cancer types; (B): TCGA analysis of SIRT1 expression in colorectal cancer, upper: COAD, lower: READ; (C): TCGA analysis of the relationship between SIRT1 expression and nodal metastasis. Upper: COAD, lower: READ; (D): TCGA analysis of the relationship between SIRT1 expression and stage classification. Upper: COAD, lower: READ; (E): Western blot detection of SIRT1 expression in 16 patient tissue samples, bottom: grayscale statistics, **P* < 0.05; (F): qRT-PCR detection of the relationship between SIRT1 expression and age, gender, T, N, M staging, **P* < 0.05, ***P* < 0.01, ****P* < 0.001.

Table S1: ShRNA Information

| Name | Sequences |
| --- | --- |
| ShA | GGGCAACACAACTATTTAT |
| ShB | GCCCTTCGAACACTAATAA |
| ShC | GTCTGTTTGCAGCCCTCTGAA |
| ShD | GTCCGTTAAGTAGCATGAAAT |

Table S2 siSIRT1 information

| gene | sense（5'-3'） | antisense（5'-3'） |
| --- | --- | --- |
| hSIRT1 si-1 | GUGCCUCAGAUAUUAAUUATT | UAAUUAAUAUCUGAGGCACTT |
| hSIRT1 si-2 | CGGGAAUCCAAAGGAUAAUTT | AUUAUCCUUUGGAUUCCCGTT |
| hSIRT1 si-3 | GGCGGCUUGAUGGUAAUCATT | UGAUUACCAUCAAGCCGCCTT |

Table S3 Antibodies information

| Antibody | Brand | Dilution ratio |
| --- | --- | --- |
| N-cadherin | CST (#13116) | 1:1000 |
| E-cadherin | CST(#3195) | 1:1000 |
| MMP9 | Proteintech (10375-2-AP) | 1:1000 |
| ZEB1 | CST (#3396) | 1:1000 |
| Snail | CST(#3879) | 1:1000 |
| vimentin | Abcam (Ab92547) | 1:1000 |
| P62 | MBL (PM045) | 1:1000 |
| LC3Ⅱ | CST (#2775) | 1:1000 |
| Beclin1 | Proteintech (11306-1-AP) | 1:1000 |
| NR3C2 | Abcam (ab64457) | 1:1000 |
| SIRT1（wb\IHC） | Abcam (ab32441) | 1:1000 |
| GAPDH | SAB (#21612) | 1:5000 |
| SIRT1（IF） | HUABIO (#M1506-3) | 1:1000 |
